# Engineering the Hole Transport Layer with a Conductive Donor–Acceptor Covalent Organic Framework for Stable and Efficient Perovskite Solar Cells

**DOI:** 10.1021/acscentsci.4c00416

**Published:** 2024-06-14

**Authors:** Shihuai Wang, Tai Wu, Jingjing Guo, Rongjun Zhao, Yong Hua, Yanli Zhao

**Affiliations:** † Yunnan Key Laboratory for Micro/Nano Materials & Technology, School of Materials and Energy, Yunnan University, Kunming 650091, Yunnan, P. R. China; ‡ School of Chemistry, Chemical Engineering and Biotechnology, Nanyang Technological University, Singapore 637371, Singapore

## Abstract

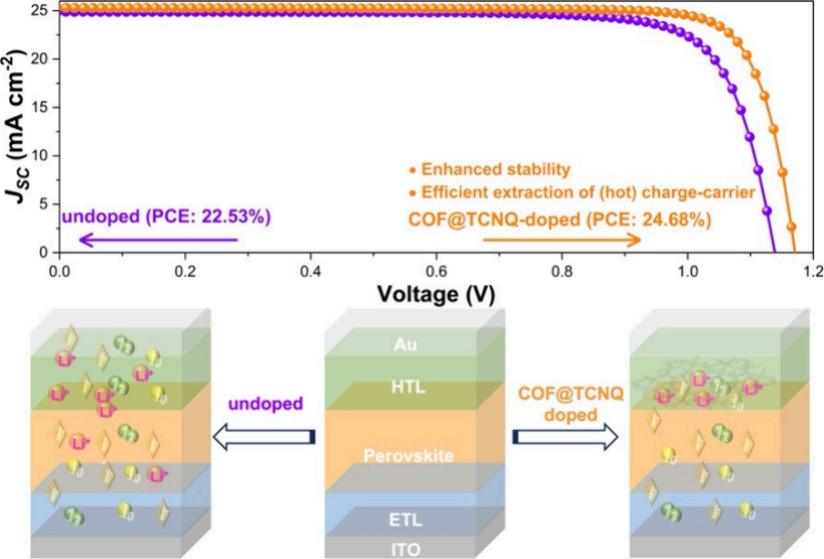

Spiro-OMeTAD doped with lithium-bis(trifluoromethylsulfonyl)-imide (Li-TFSI) and *tert*butyl-pyridine (*t*-BP) is widely used as a hole transport layer (HTL) in n-i-p perovskite solar cells (PSCs). Spiro-OMeTAD based PSCs typically show poor stability owing to the agglomeration of Li-TFSI, the migration of lithium ions (Li^+^), and the existence of potential mobile defects originating from the perovskite layer. Thus, it is necessary to search for a strategy that suppresses the degradation of PSCs and overcomes the Shockley Queisser efficiency limit via harvesting excess energy from hot charge carrier. Herein, two covalent organic frameworks (COFs) including BPTA-TAPD-COF and a well-defined donor–acceptor COF (BPTA-TAPD-COF@TCNQ) were developed and incorporated into Spiro-OMeTAD HTL. BPTA-TAPD-COF and BPTA-TAPD-COF@TCNQ could act as multifunctional additives of Spiro-OMeTAD HTL, which improve the photovoltaic performance and stability of the PSC device by accelerating charge-carrier extraction, suppressing the Li^+^ migration and Li-TFSI agglomeration, and capturing mobile defects. Benefiting from the increased conductivity, the addition of BPTA-TAPD-COF@TCNQ in the device led to the highest power conversion efficiency of 24.68% with long-term stability in harsh conditions. This work provides an example of using COFs as additives of HTL to enable improvements of both efficiency and stability for PSCs.

## Abstract 2

Covalent organic frameworks are integrated into a Spiro-OMeTAD hole transport layer to improve the photovoltaic performance and stability of solar cells for achieving a power conversion efficiency of 24.68%.

## Introduction

Hybrid organic–inorganic lead halide perovskites have shown promise as light-harvesting materials in next-generation photovoltaic (PV) technology because of their strong light absorption with narrow band gap, low-temperature solution processing, high defect tolerance, long hot-carrier diffusion lengths, and slow cooling lifetimes of the hot carriers (HCs), etc.^1−7^ While an outstanding power conversion efficiency (PCE) reaching 25.8% for state-of-the-art perovskite solar cells (PSCs) has been achieved, it is still far below the Shockley Queisser efficiency limit of ∼33% due to the current challenges of effectively collecting excess energy from hot charge carriers.^8−10^ Meanwhile, the major hindrance is their stability issue although the current efficiency of the laboratory’s cells was satisfactory enough for the practical application. In addition to rapid deactivation of the perovskite layer toward the ambient and operational conditions, the widely used hole transport layer (HTL), 2,2,7,7-tetrakis(*N*,*N*-di-*p*-methoxyphenylamine)-9,9′-spirobifluorene (Spiro-OMeTAD) with which the highest PCE has been obtained,^5,11−13^ also shows poor stability under these conditions.

The instability of Spiro-OMeTAD layer may stem from two possibilities. First, the migration of the defects or the mobile species originated from the uncoordinated Pb^2+^ and iodide species of the perovskite layer would accelerate the degradation of entire device including the HTL.^14−18^ Iodine has been considered as the most probable mobile defect as the iodide anion can readily react with interfacial charges (e.g., holes) becoming neutral atoms, which ultimately form an I_2_ molecule.^15,19^ In this regard, the density of iodine defects may increase even more under light illumination.^20^ Consequently, they would migrate rapidly across the interface, accelerating the HTL degradation. Second, owing to the low electrical conductivity and hole mobility of pristine Spiro-OMeTAD that are detrimental to interfacial charge transfer processes, Li-TFSI (lithium bis(trifluoromethanesulfonyl)imide) together with *t*-BP (*tert*-butylpyridine) as p-type dopants were added to achieve the high conductivity and hole mobility of the HTL.^21^ Yet, Li-TFSI is moisture sensitive and easily gets agglomerated, thus speeding up the degradation of the device. Furthermore, the migration of lithium cation (Li^+^) in Li-TFSI is another factor that influences the stability of the HTL and causes large hysteresis of the device.^22−24^ To improve the stability of the HTL, recent studies have focused on developing strategies to suppress the migration of lithium cations and the agglomeration of Li-TFSI. For example, Li et al. have introduced a crown ether, 12-crown-4, into the Spiro-OMeTAD precursor solution as the Li^+^ ionophore that could selectively bind Li^+^ via host–guest interaction.^12^ In this manner, Li^+^ was restrained to migrate in the HTL, thus improving the stability of the device. Additionally, some other p-type dopants^25−29^ and dopant-free hole transport materials (HTMs)^30−35^ including graphene materials and organic compounds have also been reported to solve the issues brought about by Li-TFSI as an additive. Despite these successes in suppressing the migration and aggregation of the Li-TFSI, it is obvious that mobile defects such as iodine from perovskite layer, which may also affect the stability of HTL, have rarely been considered. In order to improve the stability of the Spiro-OMeTAD based HTL as much as possible, developing materials that can not only suppress the migration and aggregation of Li-TSFI but also capture the mobile defects (i.e., iodine formed via the oxidation of uncoordinated iodides in the perovskite layer) would be highly expected.

Covalent organic frameworks are an emerging class of porous organic materials with high-ordered π−π structures, which show the excellent charge carrier mobility, infinite and discrete pore properties.^36,37^ Thus, incorporation of COFs into HTLs may hold a promise to prevent the HTL and device decomposition as the unique porous scaffold of COFs would be beneficial to capturing the mobile defects and acting as ideal platforms for Li^+^ transportation. Previously, COFs have only been employed as doping materials for PSCs to help better the crystallization of the perovskite layer,^38−43^ which facilitates the interfacial charge transfer responsible for the enhancement of PCE. However, the multifunctionality on how the COFs used in HTLs could enhance the PCE and prevent the degradation of the device has not been explored.

In this work, BPTA-TAPD COF was synthesized by reacting *N*,*N*,*N*′,*N*′-tetrakis(4-aminophenyl)-1,4-phenylenediamine (TAPD) with 2,5-bis(2-propynyloxy)terephthalaldehyde (BPTA). Then, BPTA-TAPD COF was postmodified with a well-known acceptor, tetracyanoquinodimethane (TCNQ), to prepare a well-defined donor–acceptor COF (BPTA-TAPD COF@TCNQ) with much more increased conductivity. Both COFs as multifunctional additives were subsequently incorporated into the Spiro-OMeTAD hole transport layer to fabricate corresponding PSC devices. The PSC devices with adding BPTA-TAPD COF and BPTA-TAPD COF@TCNQ exhibited enhanced photovoltaic performance and stability. The increased stability was mainly ascribed to the Li-TFSI agglomeration and the Li^+^ migration as well as mobile I^–^ defects being suppressed by BPTA-TAPD COF and BPTA-TAPD COF@TCNQ. Incorporation of BPTA-TAPD COF@TCNQ results in even more enhanced performance due to the significant enhancement of its conductivity, with which the highest PCE of 24.68% was achieved. Systematic investigations, including advanced optical and electrochemical characterizations etc., disclose that the PCE enhancement is mainly due to not only the increased extraction of the charge-carrier but also that of hot carriers.

## Results and Discussion

**Figure 1:**
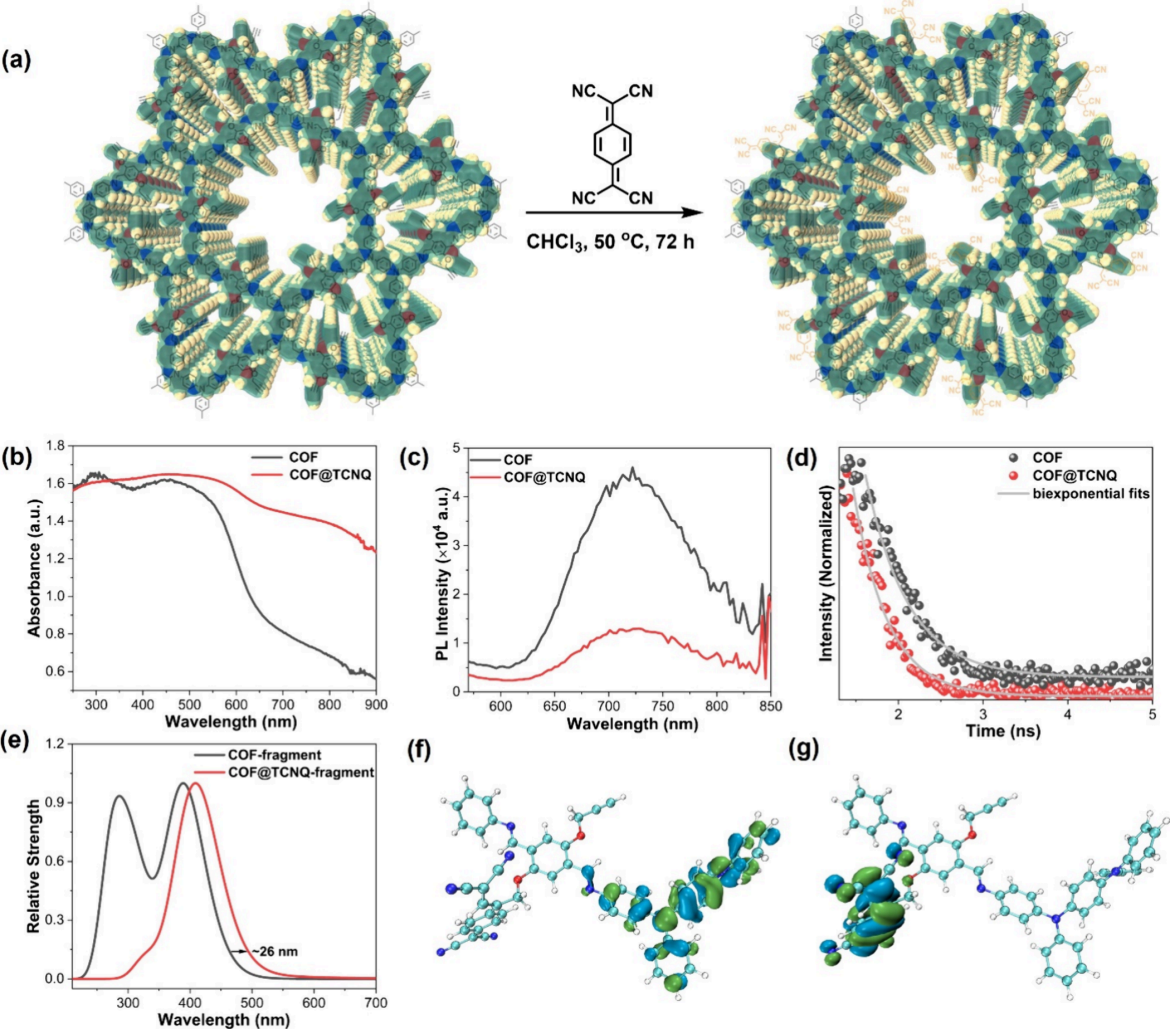
(a) Covalent TCNQ immobilization in COF through [2 + 2] cycloaddition reaction accompanied by a ring opening of the strained intermediate. (b) Diffuse reflectance UV–vis spectra, (c) PL spectra, and (d) PL decays of the parent COF and TCNQ-integrated COF. (e) Calculated UV–vis spectra of the COF-fragment and COF@TCNQ-fragment, showing the apparent red shift for the donor–acceptor complex. (f, g) Schematic illustration of the frontier orbitals in the COF@TCNQ- fragment, in which the highest occupied molecular orbital (HOMO) is calculated to be at the TAPD unit and the least unoccupied molecular orbital (LUMO) is located at the TCNQ unit.

The BPTA-TAPD COF (denotes as COF) was synthesized via an acid-catalyzed imine condensation reaction of *N*,*N*,*N*′,*N*′-tetrakis(4-aminophenyl)-1,4-phenylenediamine (TAPD) with 2,5-bis(2-propynyloxy)terephthalaldehyde (BPTA) under solvothermal conditions (see Supporting Information, SI, for the synthetic details). The TAPD moiety in this COF as an electron-donating molecule has been widely studied in solar cell and photocatalysis applications.^44−47^ Thanks to the propargyl functional groups on COF walls, a popular electron acceptor, tetracyanoquinodimethane (TCNQ), was covalently attached to the COF via [2 + 2] cycloaddition reaction of TCNQ with the ethynyl moiety. This reaction first forms a strained intermediate, which then undergoes ring opening,^48,49^ yielding the well-defined donor–acceptor BPTA-TAPD COF@TCNQ (denotes as COF@TCNQ) accompanied by a color change from red brown to dark (Figure 1a). We have proven the high crystallinity, porosity, and morphologies of the parent COF and COF@TCNQ by combining their powder X-ray diffraction, N_2_ gas sorption, and high-resolution transmission electron microscopic (HR-TEM) and scanning electron microscopic (SEM) characterizations (see SI and Figures S1–S6 for details). Spectroscopic studies including solid-state (^13^CNMR) and Fourier transform infrared (FT-IR) spectroscopies were carried out to confirm the success of covalent TCNQ immobilization in the COF. In the case of solid-state CP-MAS ^13^CNMR spectroscopic studies, we observed a significant reduction of the propargyl signals in the spectrum of TCNQ integration relative to that of the parent COF (Figure S7). The FT-IR spectra show the appearance of C≡N stretching modes at 2100 cm^–1^ for COF@TCNQ, which persisted even after a 48-h Soxhlet washing procedure with THF as the solvent (Figure S8). This is contrasted with the control experiment (simply mixing the COF with TCNQ in chloroform at room temperature) that shows no presence of TCNQ in the COF after washing, as evidenced by the FT-IR spectrum (Figure S8). These results highlight the presence of TCNQ covalently tethered into the COF. Moreover, the immense decrease of the calculated Brunauer–Emmett–Teller (BET) area from 976 m^2^/g for the parent COF to 664 m^2^/g for the COF@TCNQ suggests integration of TCNQ inside of the porous framework.

To investigate the electronic and photophysical nature of the parent COF and COF@TCNQ materials, we conducted a series of analysis including diffuse reflectance (DR) UV–vis spectroscopy, steady-state and time-resolved photoluminescence (PL), conductivity measurements, and DFT calculations. In DR UV–vis spectroscopic studies, TCNQ incorporation shows the presence of additional band resulting in an apparent red shift of the absorption profile compared with the parent COF (Figure 1b), which typically arises from intramolecular charge transfer (CT).^50,51^ The observed behavior of TCNQ-integrated COF was further validated by the steady-state and time-resolved PL spectroscopy (Figure 1c,d). In Figure 1d, the shortening of PL lifetime of the host COF can be attributed to the integration of TCNQ moieties in which CT between TCNQ and TAPD unit of the COF occurred. The averaged lifetime calculated by fitting the decay curves with biexponential function (Table S1) was 499.49 ps for the parent COF while COF@TCNQ presented shorter lifetime of 402.12 ps, indicative of a potential for CT in the COF@TCNQ system. Additionally, the electronic structure calculations also validate the donor–acceptor alignment giving rise to a red shift in optical transition after the integration of TCNQ (Figure 1e–g), which is in line with experimentally measured UV–vis spectra. The observed changes in electronic structure were further elaborated by the conductivity measurements (Figure S9), from which the conductivity of COF@TCNQ was measured to be 4.89 × 10^–4^ S cm^–1^ in comparison with the parent COF (2.35 × 10^–7^ S cm^–1^). Conductivity analysis for COF@TCNQ demonstrated a three-orders-of-magnitude increase compared with the parent COF. The band gap values estimated from Tauc plots (Figure S10) for the parent COF and COF@TCNQ were 1.90 and 1.40 eV, respectively, and they are consistent with the conductivity measurements. Similar behavior of conductivity enhancement was also reported in literature for TCNQ integration in other COF materials.^48^ The increased conductivity in COF@TCNQ may result from the formation of a charge transfer complex within COF system, which is advantageous to accelerate interfacial charge mobility and hole extraction^52,53^ when it is applied to the perovskite solar cells.

**Figure 2:**
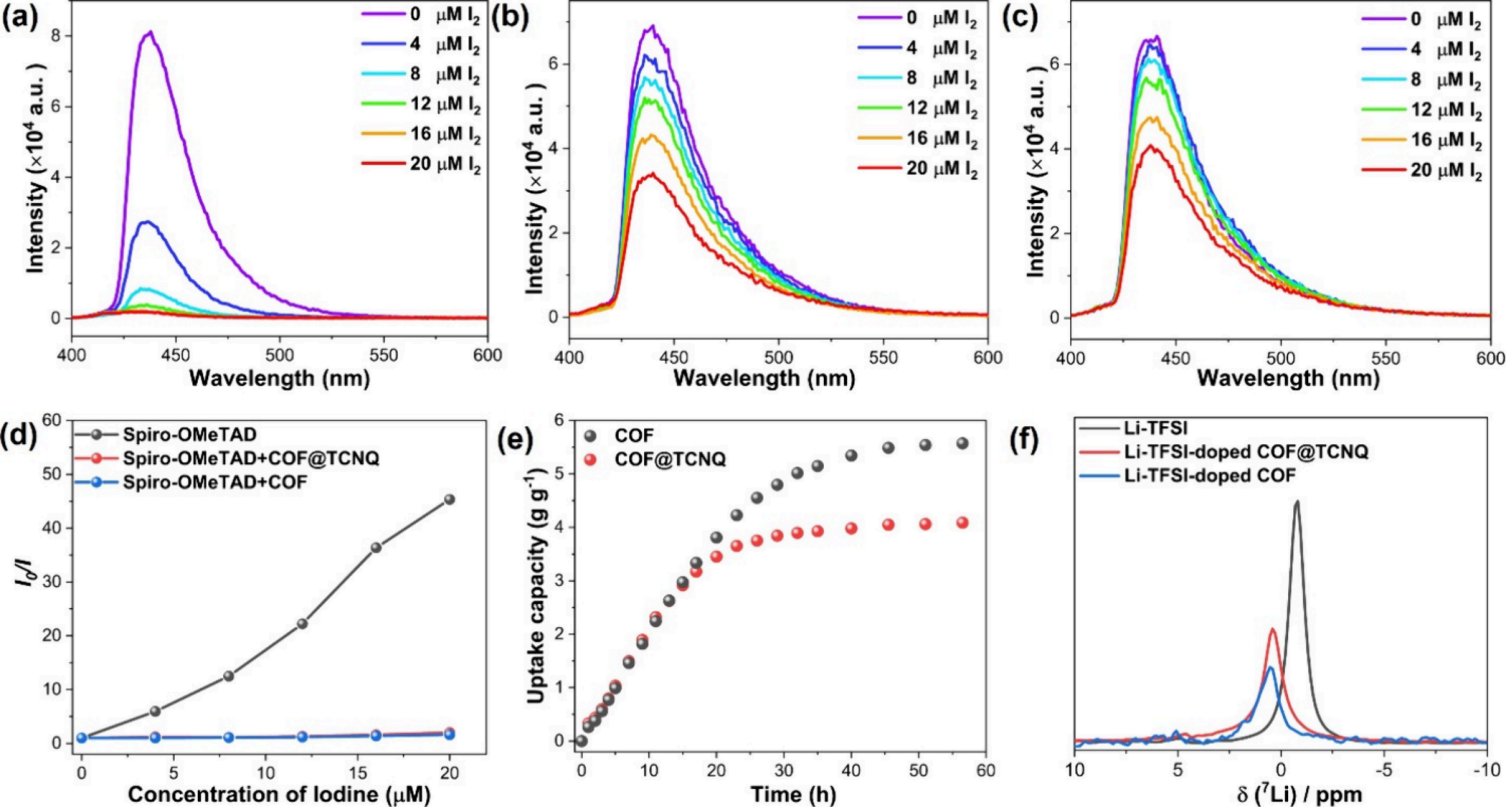
Emission quenching experiments of Spiro-OMeTAD by iodine in the absence (a) and the presence of COF@TCNQ (b) and COF (c). (d) Stern-Vomer plots of emission intensity ratio (*I*_0_/*I*) versus concentration of iodine molecule. (e) Iodine uptake of the parent COF and COF@TCNQ at 65 °C and air pressure. (f) ^7^Li NMR spectra of Li-TFSI and that after Li-TFSI uptake of the COF and COF@TCNQ.

Given that the mobile defects (i.e., iodine molecules formed), Li^+^ migration, and the agglomeration of Li-TFSI could be three of main reasons affecting the PCE and stability of PSC devices,^12,15^ we investigated the capacity of our COFs capturing iodine molecules and the ability of Li^+^ transport in porous scaffold of COFs. In Figure 2a, the significant emission quenching of the excited state of Spiro-OMeTAD with varying concentrations of iodine demonstrated the interaction of iodine with Spiro-OMeTAD. This may cause the deactivation of Spiro-OMeTAD serving as hole transport material (HTM) in the PSC device.^54^ This emission quenching by iodine titration was dramatically reduced in the presence of the COF or COF@TCNQ (Figure 2b, c). The quenching efficiencies of the three cases were fitted following the Stern–Volmer expression:^55^
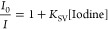
1where *I*_0_ and *I* refer to the emission intensities without and with iodine quencher, respectively; *K*_SV_ represents the Stern–Volmer constant, and [Iodine] is the concentration of the iodine quencher. From the Stern–Volmer plots (Figure 2d), in the absence of COF@TCNQ or COF, much more efficient quenching of the excited state of Spiro-OMeTAD by iodine titration is probably assigned to effective electron transfer between iodine and Spiro-OMeTAD or the heavy atomic effects of the iodine, which deactivates the function of Spiro-OMeTAD as HTM. We then explore iodine vapor adsorption by exposing COFs to iodine vapor at ambient pressure and 65 °C (Figure 2e). Both the parent COF and COF@TCNQ exhibited rapid iodine uptake in the first 15 h, and then reached saturated adsorption in 25 and 40 h, respectively. The maximum capacity of iodine uptake over 60 h was calculated to be 5.51 g g^–1^ for the parent COF and 3.62 g g^–1^ for COF@TCNQ. Thus, for above titration experiments of iodine in the presence of COF or COF@TCNQ (Figure 2b, c), the suppressed emission quenching of the excited state of Spiro-OMeTAD could be attributed to iodine capture of COFs that may retain the function of Spiro-OMeTAD as HTM in the PSC device. To test the interaction of Li-TFSI with COFs, we conducted Li-TSFI adsorption experiments by immersing the parent COF and COF@TCNQ in an acetonitrile solution of Li-TSFI at room temperature for 24 h (see SI for details). Figure 2f shows ordinary ^7^Li NMR spectra of the pure Li-TSFI and Li-TFSI adsorbed COFs. Compared with pure Li-TFSI, the apparent chemical shift in Li-TFSI adsorbed COF or COF@TCNQ demonstrated Li^+^ transport in the porous scaffold of COFs. Previous studies have also shown that COF materials enable the lithium-ion conduction through the porous channels that aid in ionic bond dissociation, thus providing a pathway for Li^+^ transport.^56−58^

Next, we investigate how the perovskite/HTL films benefit from the addition of COF and COF@TCNQ. Figure S11 shows the scanning electron microscopic (SEM) morphology of the perovskite lattice in which some defects can be found on the surface. Particularly, oxidation chemistry of iodide defects by interfacial charges forming iodine molecules may migrate across interface and damage the HTL, as discussed above. Spiro-OMeTAD precursor solutions without and with COFs doping were spin-coated onto perovskite layer to fabricate the HTL films for characterizations (SI for the detailed procedure). For the freshly prepared films, there is no apparent difference in surface morphologies among the control, COF-doped, and COF@TCNQ-doped HTL films (Figure S12) while the control film exhibited less stability after about one month in comparison with COF- and COF@TCNQ-doped HTL films (Figure S13). Figure S13a shows the SEM morphology and EDX mapping of the control film stored in ambient conditions for 35 days, where we observed the agglomeration of Li-TFSI located at the edge of the film owing to Li^+^ migration. By contrast, the SEM morphologies and EDX analysis of the COF- and COF@TCNQ-doped films manifested that Li-TFSI was uniformly distributed under the same conditions (Figure S13b, c). Thus, COF- and COF@TCNQ-doped HTL films greatly retained the surface morphologies because of Li^+^ ions transport in porous scaffold of COFs that prevents their agglomeration on the HTL (Figure 2f). Atomic force microscopy (AFM) studies were also conducted to explore the effect of the addition of COFs on surface morphology of Spiro-OMeTAD HTL films. Figure S14 presented AFM images of the control, COF-doped, and COF@TCNQ-doped HTL films. The root-mean-square (RMS) roughness of the control film is 14.5 nm, which is decreased to 8.66 nm for COF@TCNQ-doped film and 10.6 nm for COF-doped film. This trend indicates enhanced compactness for COFs-doped HLT films, which is beneficial to obtain high-performance PSC devices.^25^

**Figure 3:**
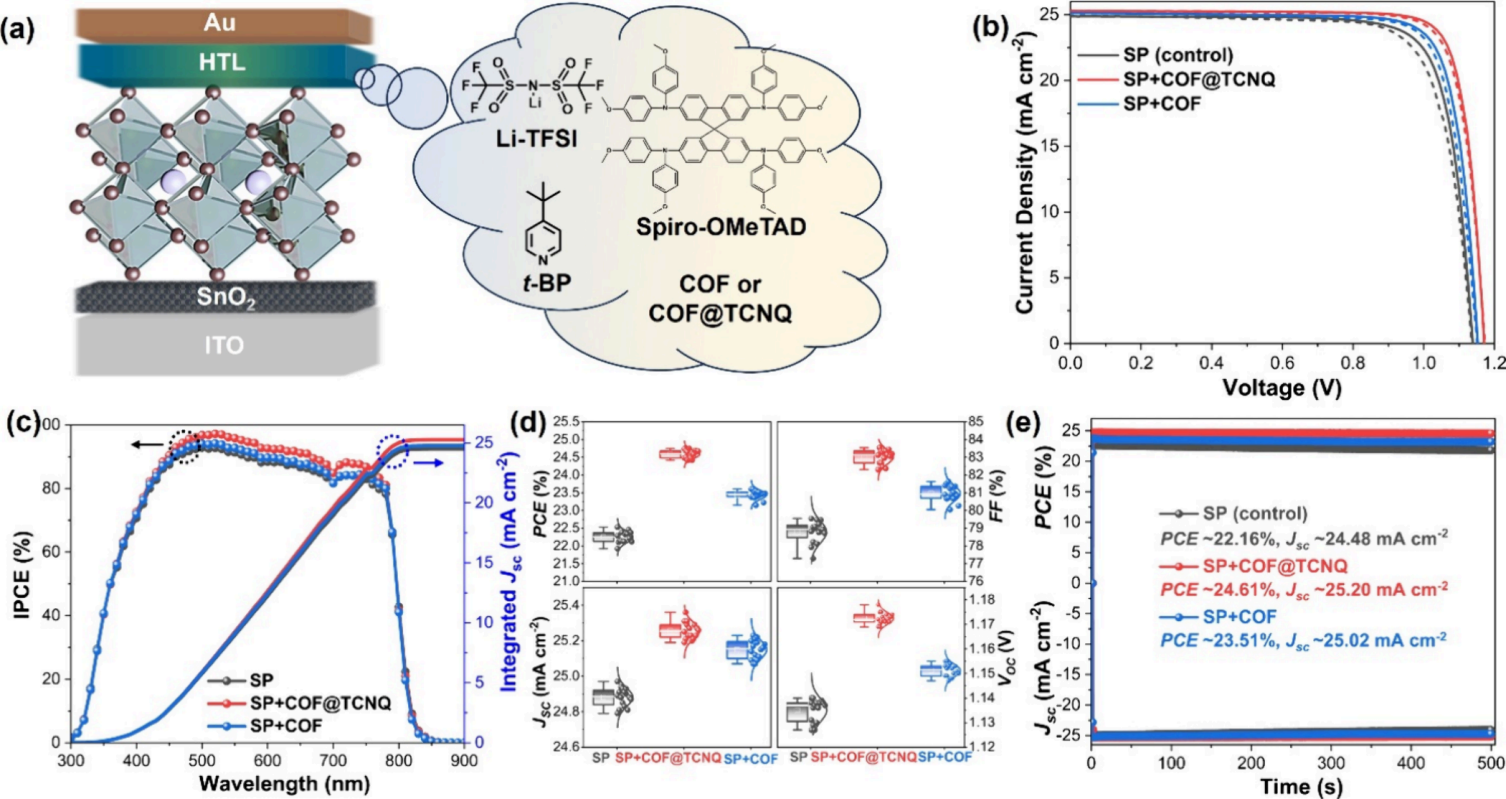
(a) Schematic representation of the device configuration showing the incorporation of COF@TCNQ or COF into the Spiro-OMeTAD layer. (b) *J–V* curves of the control, COF@TCNQ-, and COF-treated devices measured under reverse scanning (solid lines) and forward scanning (dashed lines) modes. (c) IPCE spectra and integrated *J*_SC_ characteristics of the devices based on the control, COF@TCNQ-, and COF-doped Spiro-OMeTAD layer. (d) Statistical distributions of the photovoltaic parameters of 16 cells fabricated from each type of device. (e) Stabilized PCE outputs of the control, COF@TCNQ- and COF-treated devices over time. Note: Spiro-OMeTAD in the graphic, if not stated otherwise, was further abbreviated as SP in this manuscript due to the limited space.

These effects of doping COF or COF@TCNQ into Spiro-OMeTAD HTL were then reflected in the photovoltaic performance of PSC devices, and the PSC possessing a device architecture of ITO/SnO_2_/perovskite/Spiro-OMeTAD without and with COF or COF@TCNQ/gold was fabricated via two-step method (Figure 3a): the perovskite composite is FAPbI_3_ doping with a small amount of MACl and MABr as additives, where FA and MA stand for formamidinium and methylammonium, respectively. Experimental details of the cell fabrication were presented in the SI. The content of the parent COF and COF@TCNQ doped in Spiro-OMeTAD layer was optimized, and in our case 5 mL COF (2 mg/mL) and 7 mL COF@TCNQ (2 mg/mL) solutions as optimal conditions were used to obtain the best photovoltaic performance of the corresponding devices (Figures S15 and S16). Figure 3b shows the current density–voltage (*J–V*) characteristic curves of the PSCs based on the control, COF@TCNQ, and COF doped Spiro-OMeTAD HTL under AM 1.5G illumination. According to the *J–V* curves of these PSC devices, the corresponding parameters of photovoltaic performance were then summarized in Table 1. The undoped PSC device exhibits a short-circuit current density (*J*_SC_) of 24.89 mA cm^–2^, an open-circuit voltage (*V*_OC_) of 1.138 V, and fill factor (FF) of 79.55%, resulting in a PCE of 22.53% during reverse scanning. The PCE was improved to 23.60% combining with a *V*_OC_ of 1.153 V, a *J*_SC_ of 25.14 mA cm^–2^ and an FF of 81.41% when using COF doped Spiro-OMeTAD HTL. In the device with COF@TCNQ doped Spiro-OMeTAD HTL, the PCE was further enhanced to 24.68% with a much improved *J*_SC_ of 25.29 mA cm^–2^, *V*_OC_ of 1.171 V and FF of 83.34%.

**Table 1:** Photovoltaic Parameters of the Devices^a^

cell	scan mode	*V*_OC_ (V)	*J*_SC_ (mA cm^–2^)	FF (%)	PCE (%)	HI (%)
**SP**	reverse	1.138	24.89	79.55	22.53	3.1
	forward	1.136	24.95	77.05	21.84	
**SP+COF@TCNQ**	reverse	1.171	25.29	83.34	24.68	1.2
	forward	1.173	25.27	82.27	24.38	
**SP+COF**	reverse	1.153	25.14	81.41	23.60	1.9
	forward	1.150	25.10	80.19	23.15	

a HI indicates the hysteresis of the devices under reverse scanning and forward scanning.

Due to its high hole mobility and efficient charge-carrier extraction arising from the superior conductivity of the donor–acceptor COF,^48^ the PCE of COF@TCNQ treated device has increased about 10% in comparison with the control device. It is also disclosed that the control device shows a relatively large PCE difference under reverse scanning (RS) and forward scanning (FS), as shown in Table 1. On the contrary, a smaller hysteresis is found in the *J–V* curves of the devices based on COF- and COF@TCNQ-doped Spiro-OMeTAD HTLs, giving PCEs of 23.15% (*J*_SC_ = 25.10 mA cm^–2^, *V*_OC_ = 1.150 V, and FF = 80.19%) and 24.38% (*J*_SC_ = 25.27 mA cm^–2^, *V*_OC_ = 1.173 V, and FF = 82.27%) under FS, respectively. The reduced hysteresis for COF@TCNQ and COF treated devices might be associated with the presence of COF and COF@TCNQ suppressing the migration of Li^+^ ions.^12,24^ The integrated *J*_*sc*_ values calculated from the incident photon-to-electron conversion efficiency (IPCE) spectra are 24.50 mA cm^–2^ for the control device, 25.22 mA cm^–2^ for the COF@TCNQ treated device, and 24.81 mA cm^–2^ for the COF treated device (Figure 3c), which match well with the measured *J*_SC_. Figure 3d shows the statistical distributions of the photovoltaic performance parameters of 16 cells fabricated from each type of device, from which both COF and COF@TCNQ treated devices have better reproducibility than the control one.

To understand the operating state of these devices, the stabilized photocurrent outputs and efficiency are measured at the maximum power point (MPP). Under continuous MPP for 500 s, the initial PCE has only lost about 1% for COF@TCNQ treated device and 2% for COF treated sample but the control sample dropped to 92% (Figure 3e). To verify the universality of COF@TCNQ as an efficient additive for hole transport materials (HTMs), we also prepared the PSC device using well-known HTMs of X60. Figure S17 shows the best *J–V* characteristic curves of PSCs, and the corresponding photovoltaic parameters are summarized in Table S2. Compared to 21.89% PCE (*J*_SC_ = 24.72 mA cm^–2^, *V*_OC_ = 1.142 V, and FF = 77.56%) for the control X60 device, the device with X60+COF@TCNQ as the HTL exhibits an enhanced PCE value of 24.03% with enhanced *V*_OC_ of 1.171, *J*_SC_ of 24.87, and FF of 82.51% and negligible hysteresis under reverse scanning.

**Figure 4:**
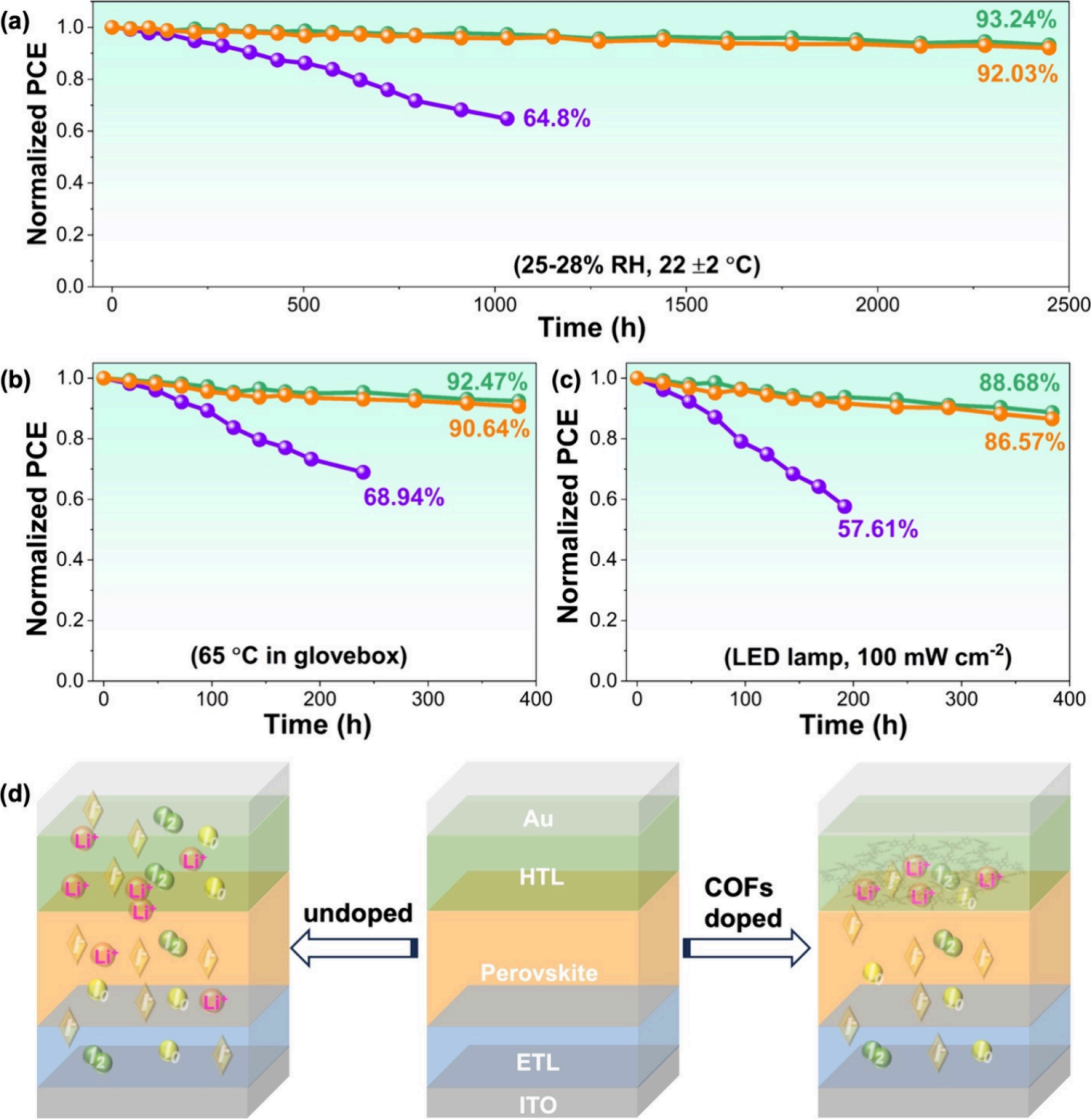
Shelf stability of the control, COF@TCNQ- and COF-treated devices under ambient condition with RH of 25−28% (a), heating at 65 °C (b) and soaking with LED light in glovebox (c). The purple, cyan, and orange lines correspond to the PSC devices based on the control, COF@TCNQ-, and COF-doped Spiro-OMeTAD layers, respectively. (d) Schematic illustration of mobile defects and Li^+^ migration being suppressed by addition of COF@TCNQ or COF into Spiro-OMeTAD layer.

The long-term stability of unencapsulated samples was then investigated by storing them under a relative humidity (RH) of ∼28% at room temperature. Figure 4a shows that the COF and COF@TCNQ doped samples retained 92.03% and 93.24% of their original efficiencies in a 2450-h monitoring period, respectively. In contrast, the control sample showed significant decrease of the PCE, and only 64.8% of the original value was preserved after 1000 h. The thermal stability was also explored by storing these unencapsulated cells in a N_2_-filled glovebox at 65 °C. In Figure 4b, the PCE of the control cell rapidly degrades to 68.94% of its original value. In comparison, the unencapsulated devices based on COF- and COF@TCNQ-doped Spiro-OMeTAD displayed enhanced thermal stability, and could maintain 90.64% and 92.47% of their original efficiencies after 384 h, respectively.

In addition, photostability test results in Figure 4c show that the COF@TCNQ and COF treated cells retained over 86% of their initial efficiencies after being soaked continuously with a white light-emitting diode (LED, 100 mW/cm^2^) for 384 h in a N_2_-filled glovebox. Yet, the control cell dropped to ∼57.61% of its initial PCE in 200 h. These results demonstrate that COF and COF@TCNQ as additives of Spiro-OMeTAD can be a useful strategy to enhance the stability of the cell under ambient, thermal, and light soaking conditions. Particularly, with thermal and light-illumination conditions, the formation of potential mobile defects and rapid Li^+^ migration would accelerate the degradation of the control PSC device. As illustrated in Figure 4d, the presence of the COF or COF@TCNQ in the device may be able to not only capture the mobile defects but also suppress the agglomeration of Li-TFSI, thus enhancing the stability under these conditions. Note that the existence of COF and COF@TCNQ also could render exceptionally hydrophobic HTL, which blocks H_2_O/O_2_ penetration/erosion and thus prevent rapid degradation of the device.^7,25^ Thus, the water contact angles of the control, COF@TCNQ-, and COF-treated PSC devices were measured (Figure S18). As expected, COF@TCNQ- and COF-treated PSC devices with water contact angles of 91° and 90°, respectively, are more hydrophobic compared to that observed from the pristine film (water contact angle = 61°). The increased hydrophobicity in the COF@TCNQ- and COF-treated samples could prevent the Li-TFSI being exposed to moisture air, leading to the improved stability of perovskite device.

To address the improved performance of the PSC devices with COF- and COF@TCNQ-doped Spiro-OMeTAD HTLs, comprehensive analysis revealing charge-carrier dynamics was employed. We performed the conductivity measurements of HTL-only films since it is a very important parameter to facilitate the mobility and extraction of the holes leading to highly efficient PSCs. The conductivity of the control film was calculated to be 6.20 × 10^–5^ S cm^–1^, which is increased to 3.41 × 10^–4^ S cm^–1^ for the COF@TCNQ-doped HTL film and 8.73 × 10^–5^ S cm^–1^ for the COF-doped HTL film (Figure S19). Here, the significant increase of the conductivity in COF@TCNQ-doped HTL film is ascribed to the excellent conductivity of COF@TCNQ arising from the formation of a charge transfer complex in the well-defined donor–acceptor COF material. This conductivity enhancement is in line with conductivity measurements of the COF materials described above, and is highly desirable to accelerate the hole mobility.^59^ Thus, we utilized the space-charge-limited-current (SCLC) technique to probe the hole mobility at perovskite/HTL interface, and a hole-only device with a structure of ITO/PEDOT: PSS/perovskite/COFs doped Spiro-OMeTAD or undoped Spiro-OMeTAD/Au was prepared for this investigation.

**Figure 5:**
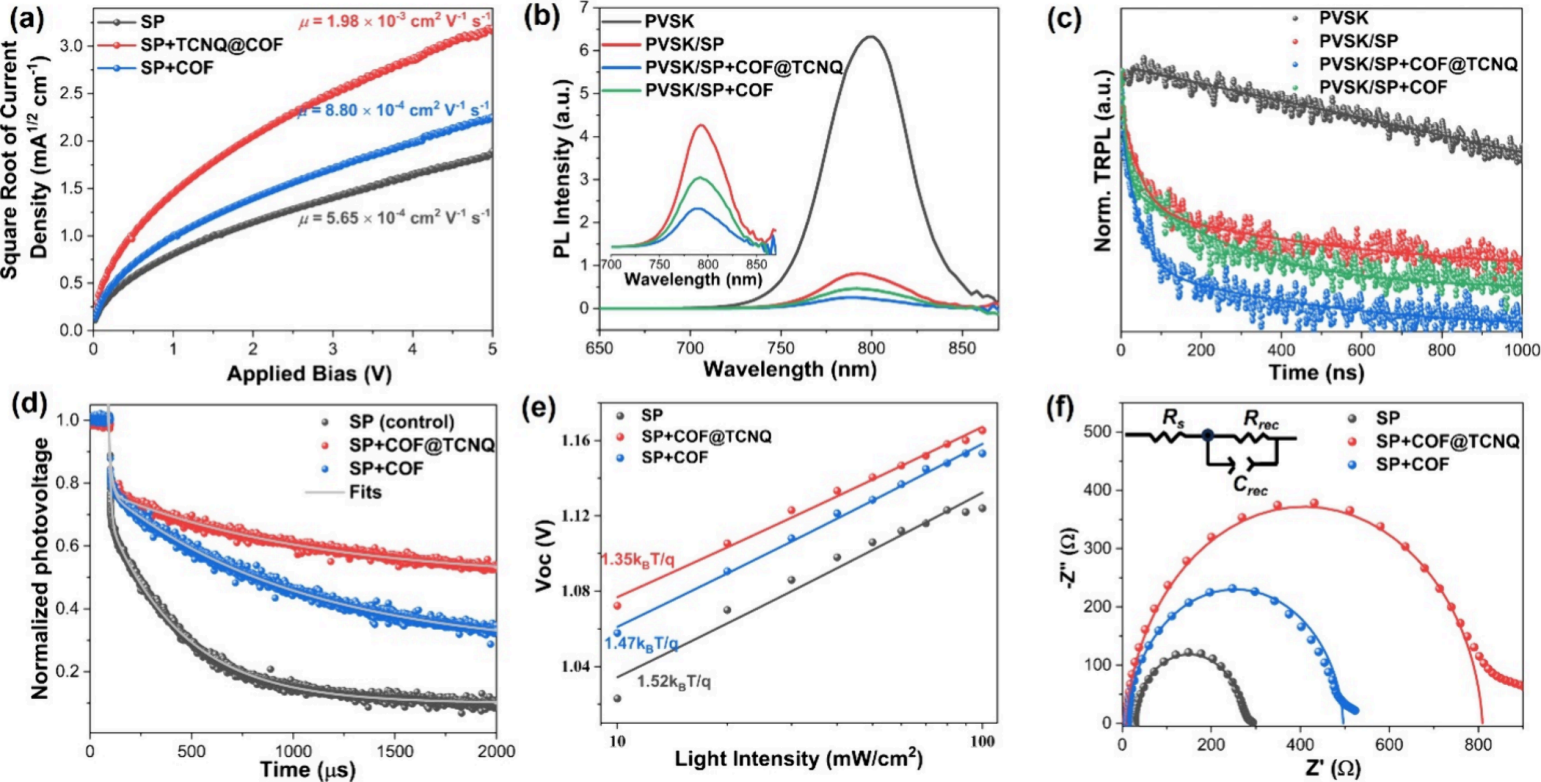
(a) *J–V* curves of hole-only devices based on ITO/PEDOT: PSS/perovskite/HTL/Au architecture (HTL = Spiro-OMeTAD or COF@TCNQ doped Spiro-OMeTAD or COF doped Spiro-OMeTAD). (b) PL spectra and (c) time-resolved PL plots of ITO/perovskite/HTL films showing the quenching of the excited state of perovskite in the presence of HTL. PVSK defined in the graphic stands for perovskite, and is applied thereafter. (d) Transient photovoltage decays, (e) plots of *V*_OC_ versus light intensity and (f) Nyquist plots of the control, COF@TCNQ-, and COF-treated samples.

As shown in Figure 5a, the hole mobility (m) was estimated to be 5.65 × 10^–4^ cm^2^/V·s for the control film, 2.98 × 10^–3^ cm^2^/V·s for the COF@TCNQ-doped HTL film, and 7.80 × 10^–4^ cm^2^/V·s for the COF-doped HTL film. These results demonstrated a small increase in the hole mobility for COF-doped HTL film versus control film, while the COF@TCNQ-doped film displayed tremendously improved hole mobility resulting from the superior conductivity of the donor–acceptor COF material. The hole mobility is closely correlated to the hole trap-state density (*N*_t_). The hole *N*_t_ was then examined by the SCLC model (Figure S20), from which the *N*_t_ was estimated according to the following equation, *N*_t_ = 2ε_0_εV_TFL_/*eL*^2^,^60^ where *V*_TFL_ is the onset voltage of the trap-filled limit area, *L* is the thickness of the device, and *e* is an elemental charge. The calculated *N*_t_ values were given in the SI (Table S3). We observed a significant decrease of trap density for COF@TCNQ doped Spiro-OMeTAD HTL. Taken together with the accelerated hole mobility and the reduced hole trap density at perovskite/Spiro-OMeTAD+COF@TCNQ interface, we proposed that an efficient hole extraction, which is conducive to achieving high-performance PSCs, would be expected in the COF@TCNQ treated device.

To shed light on this hypothesis of efficient hole extraction for COF@TCNQ doped HTL, we investigated the energy level alignment of HTL films by using UV–Vis spectroscopy and ultraviolet photoelectron spectroscopy (UPS). The optical bandgaps estimated from Tauc plots are found to be 3.00, 2.99, and 2.97 eV for the control, COF@TCNQ, and COF-treated Spiro films, respectively (Figure S21). Furthermore, the work functions (*W*_f_) estimated from UPS data are 4.35, 4.78, and 4.66 eV for the Spiro-OMeTAD, Spiro-OMeTAD+COF@TCNQ, and Spiro-OMeTAD+COF films, respectively (Figures S22–S24). Thus, their Fermi level (*E*_f_) is −4.35, −4.78, and −4.66 eV, respectively. It can be clearly seen that the introduction of COF@TCNQ can enhance the Fermi level of Spiro-OMeTAD-based HTL. The HOMO energy level (*E*_HOMO_) of the films can be estimated by the following expression: *E*_HOMO_ = *E*_f_ – *E*_onset_.^61^ Thus, the band structures of these films are aligned. As shown in Figure S25, Spiro-OMeTAD+COF@TCNQ film exhibits more favorable energy level alignment with the perovskite material, which is beneficial to extract and transport the holes from the perovskite layer to HTL for enhancing *V*_OC_ in the device. Moreover, the charge-carrier transport dynamics at the perovskite/HTL interface were examined using steady-state photoluminescence (PL) and time-resolved photoluminescence (TRPL) spectroscopic techniques. Due to the presence of the Spiro-OMeTAD HTM, the PL intensity of the perovskite (Figure 5b) was drastically quenched. Both COF- and COF@TCNQ-doped devices showed stronger PL quenching of the perovskite emission with respect to undoped perovskite/Spiro-OMeTAD film, suggesting more efficient charge-carrier extraction in the COF- and TCNQ-doped Spiro-OMeTAD HTL. The TRPL decays of the four samples together with their biexponential fits were shown in Figure 5c. The fitted lifetimes of these samples were summarized in Table S4, SI. In the perovskite-only device, the TRPL decay corresponds to the recombination of electron–hole pair with an average lifetime of 2237.66 ns. For perovskite/HTL samples, the fitted average lifetimes of the decays were decreased to 189.13 ns for perovskite/Spiro-OMeTAD film, 107.93 ns for perovskite/Spiro-OMeTAD+COF@TCNQ film, and 163.22 ns for perovskite/Spiro-OMeTAD+COF film. Our observations tentatively demonstrate that COF@TCNQ doped Spiro-OMeTAD may offer a better capability to extract the photogenerated carriers (i.e., holes) from perovskite layer than the control and COF treated samples. However, in some cases, the quenching of perovskite PL signals by accelerated charge recombination and by charge transfer at perovskite/HTL interface as well as by other processes could not be explicitly distinguished.^62^

Thus, the transient photovoltage (TPV) experiments illustrating charge carrier recombination were carried out. In Figure 5d, the TPV decays imply the recombination rates of the charge carrier. Typically, rapid TPV decay corresponds to fast recombination that is unfavorable to the PCE of the device.^27^ The charge carrier lifetimes of the control, COF@TCNQ-, and COF-treated samples were calculated with a single exponential fit as 318 ms, 787 ms, and 590 ms, respectively. The slower recombination rate of charge carrier in the COF@TCNQ treated device thus confirms efficient charge-carrier extraction from perovskite layer to HTL, which enables the observation of the significantly enhanced *V*_OC_ and FF. To gain more insights regarding the carrier recombination, we conducted the light intensity (*I*_light_)-dependent *V*_OC_ characterizations of these cells since the increased *V*_OC_ in the device correlates closely with suppressing recombination process.^63^ The ideality factor (*n*_id_) proportional to *V*_OC_ was calculated to be 1.52, 1.35, and 1.47 for the control sample, COF@TCNQ treated film, and COF treated device (Figure 5e), respectively. The details of *n*_id_ calculations were presented in the SI. Obviously, a smaller *n*_id_ observed in the COF@TCNQ treated device indicates that the interfacial trap-assisted recombination can be greatly suppressed. The charge transport in turn should be sped up in the COF@TCNQ treated device. The overall trends here mirror the hole mobility and trap density values determined by the SCLC measurements. In summary, the COF@TCNQ doped Spiro-OMeTAD here could act as an efficient HTM with excellent capability of hole extraction.

We therefore utilized electrochemical impedance spectroscopy (EIS) to study the interfacial charge transport. EIS measurements were conducted under dark conditions at a bias of 0.1 V. The EIS data were then fitted using a simple equivalent circuit model, which consists of series and parallel resistance elements as well as a parallel capacitance (Figure 5f). The fitted results show that the series resistance (*R*_s_) is <8 Ω cm^2^ for all devices. The parallel resistance here indicates the recombination resistance. The recombination resistance (*R*_rec_) correlating to the diameter of the major semicircular characteristic varies from 231 and 484 Ω cm^2^ for undoped Spiro-OMeTAD and COF doped Spiro-OMeTAD, respectively, to 813 Ω cm^2^ for COF@TCNQ doped Spiro-OMeTAD. The increase in *R*_rec_ with COF- and COF@TCNQ-doped Spiro-OMeTAD HTL associates with the improvements of *V*_OC_ and FF, and proves that hole extraction from the perovskite layer is more efficient for Spiro-OMeTAD HTL treated with COF and COF@TCNQ.

**Figure 6:**
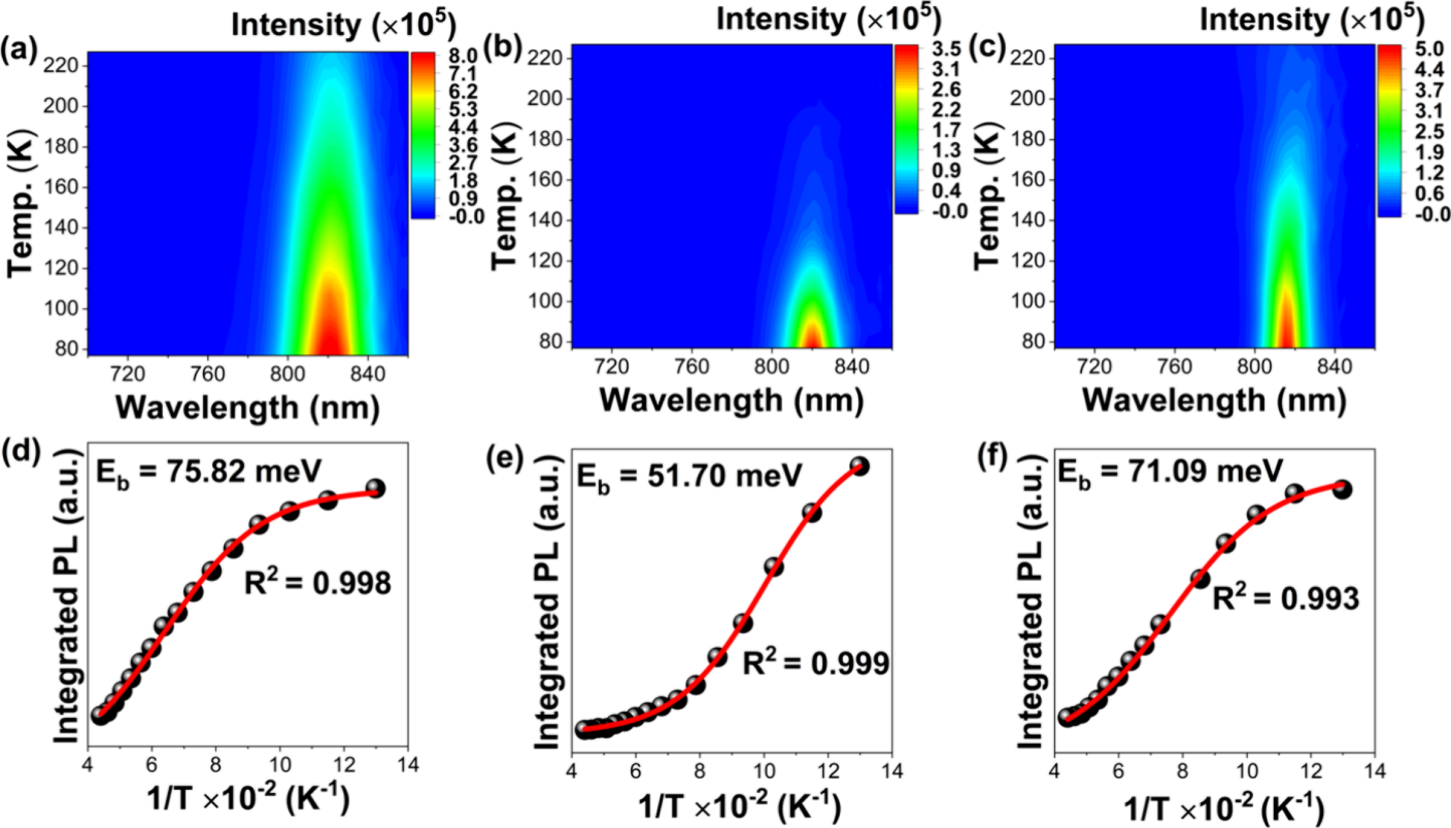
Temperature-dependent pseudocolor 2D PL plots (top) and Arrhenius plots (bottom) determining exciton binding energies. (a, d) Control device, (b, e) COF@TCNQ treated sample, and (c, f) COF treated device.

COF@TCNQ doped Spiro-OMeTAD HTL leading to much more efficient hole extraction from perovskite film was further studied by the temperature-dependent PL spectra measurements that estimates the exciton binding energies upon excitation of perovskite film. Following excitation at 470 nm (Figure 6), the steady-state PL spectra of the control, COF@TCNQ-, and COF-treated samples were recorded at a temperature range of 77 to 223 K. The spectra show a broad maximum at ∼820 nm, which is in accordance with ones measured in room temperature (Figure 5b). The PL intensity for all samples gradually decreased with increasing temperature (Figure 6a–c), strongly indicative of photoinduced exciton generation being thermally activated.^64,65^ Compared to the control sample, we also observed an accelerated decrease of PL intensity for the COF@TCNQ and COF treated films, indicating that the charge carrier can be rapidly extracted once the photogenerated exciton is thermally activated. Hence, the exciton binding energies (*E*_b_) are determined by fitting the integrated PL data points according to eq 2:^65^
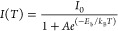
2where *I*_0_ is the PL intensity at low temperature, *k*_B_ is the Boltzmann constant and *A* is a proportional constant. From the fitting, the *E*_b_ values for the control, COF@TCNQ-, and COF-treated films were found to be 75.82 meV, 51.70 meV, and 71.09 meV, respectively (Figure 6d–f). The *E*_b_ is essentially reduced for Spiro-OMeTAD HTL treated with COF and COF@TCNQ. In general, lower *E*_b_ indicates the enhanced distance between photogenerated electron and hole, which facilitates the dissociation of excitons into free charge carriers. In our case, the decreased *E*_b_ for COF@TCNQ and COF treated samples demonstrate that there is an accelerated charge transfer to occur, thus enhancing the ratio of free carriers. Particularly, the large decrease of *E*_b_ in the COF@TCNQ doped Spiro-OMeTAD sample suggests the increased ratio of free carriers due to superior hole extraction from perovskite film.

**Figure 7:**
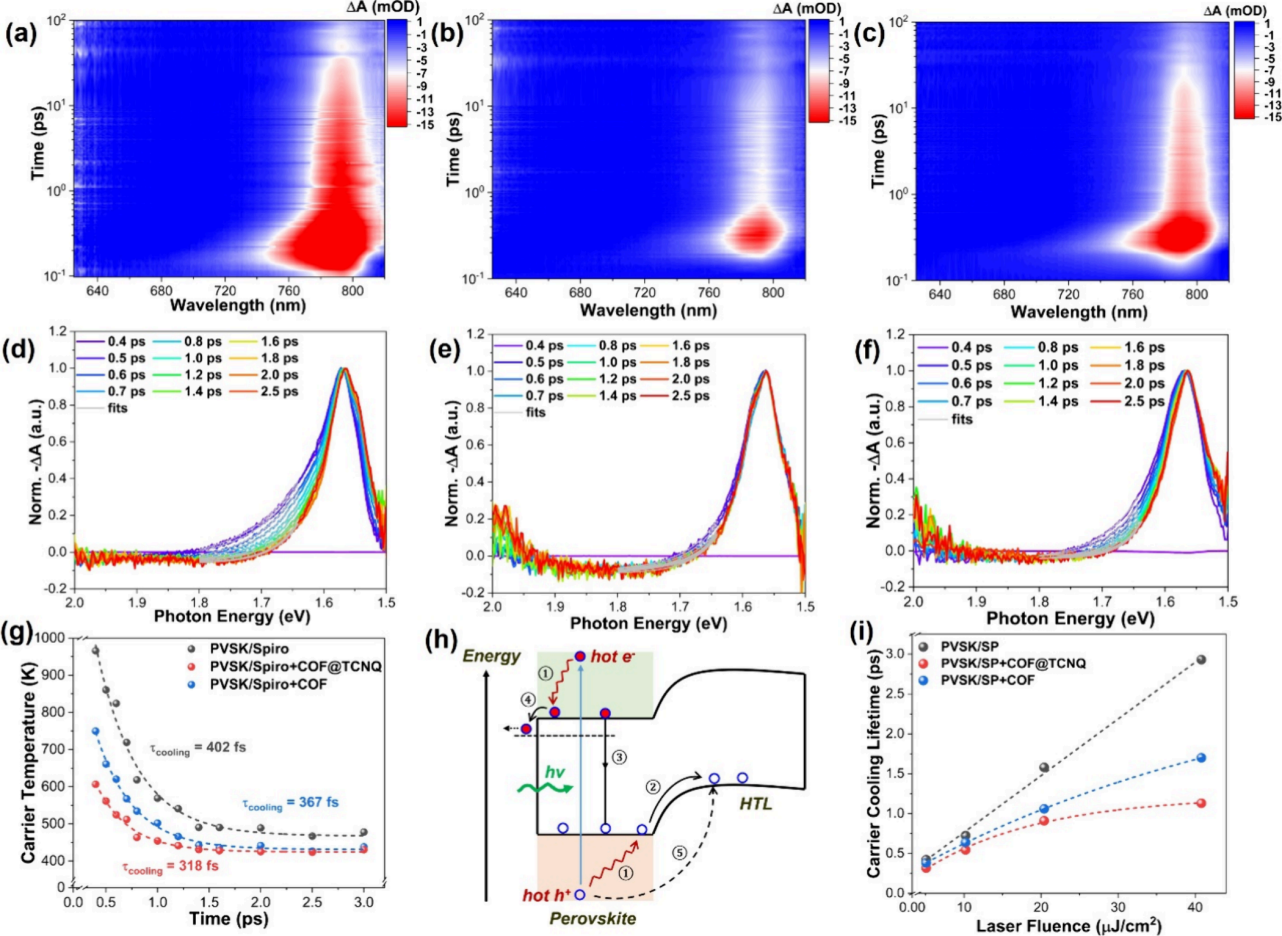
Pseudocolor 2D TA data (top panel) and the corresponding normalized TA spectra (middle panel) pumped at a photon energy of 3.10 eV. (a, d) Perovskite/Spiro-OMeTAD, (b, e) perovskite/COF@TCNQ-doped Spiro-OMeTAD, and (c, f) perovskite/COF-doped Spiro-OMeTAD. (g) Plots of hot-carrier temperature versus delay time for the perovskite/Spiro-OMeTAD, perovskite/COF@TCNQ-doped Spiro-OMeTAD, and perovskite/COF-doped Spiro-OMeTAD films with low pump fluence of 5.1 μJ cm^–2^. (h) Schematic representation of hot-carrier (HC) dynamics of the device upon photoexcitation: ① HC (including hot electrons and holes) cooling; ② hole transfer from the band edge of the perovskite to HTL; ③ exciton recombination process; ④ injection of electrons to electron transport layer; ⑤ the extraction process of hot holes at deeper energy state. (i) Plots of HC cooling time as a function of pump fluence for the three samples, where slow increase of HC cooling time with increasing pump fluence suggests the capability of extracting hot holes from perovskite layer in the COF@TCNQ- and COF-treated samples.

In all PSC devices, incident photons (for example by the sunlight irradiation) having energies higher than the bandgap of perovskite semiconductor could create electron–hole pairs (excitons) or charge carriers with excess energies above the band edge, which are called “hot excitons or hot carriers” (HCs).^8,66^ The HCs can then cool down to the lattice temperature or relax to the band edge along with the loss of their excess energies to phonons. This energy loss might be regarded as one of the main loss pathways for photovoltaic efficiency in PSC devices.^8^ Thus, accelerating HCs extraction for the effective collection of HCs energy before relaxing to the band edge of the perovskite semiconductor enables enhanced PCE of a single-junction PSC, allowing PCE to increase to the thermodynamic calculation of ∼66%.^67^ To explain the HCs extraction, we conducted femtosecond transient absorption (TA) spectroscopic measurements, in which we dedicate ourselves to illustrating the fate of the HCs at very early delay times in the sample when Spiro-OMeTAD HTL was treated with COF or COF@TCNQ. Upon excitation with a pump photon energy of 3.10 eV, the 2D pseudocolor TA plots and the corresponding normalized TA spectra of the three samples studied here were compared at pump fluence of 5.1 μJ/cm^2^ (Figure 7). At early delay times, we observed a negative (Δ*A* < 0) band centered around the bandgap of ∼1.55 eV for all samples. This can be ascribed to the ground-state photobleaching (PB) induced by band-edge filling of carriers,^10,67^ which agrees undoubtedly with their steady-state absorption spectra (Figure S26).

Furthermore, the PB signal in the normalized TA spectra shows a broadening phenomenon toward the high energy tail (beyond the bandgap of 1.55 eV) at a very early delay-time window. This occurrence in the normalized TA spectra results from the quasi-equilibrium distribution of the HCs, which is characterized by a carrier temperature (*T*_c_). As illustrated in Figure 7a–f, the broadening event of the high energy tail (HET) gradually narrows with a time window of 0.4–3.0 ps, corresponding to the HCs cooling processes (for instance, cooling of hot electrons from a higher conduction-band state CB2 to the band edge CB1, or cooling of hot holes from a deeper valence-band state VB1 to the band edge VB2). It disappears faster in the COF@TNCQ and COF treated samples in comparison with the control film, demonstrated as a more rapid cooling process of the HCs for COF@TNCQ and COF treated samples, which may benefit from Li^+^ migration and mobile defects being suppressed by COF@TCNQ or COF to increase the capability of the HCs extraction. Because the Δ*A* correlates directly with Fermi–Dirac distributions of the HCs, the HET observed here could be described by the Maxwell–Boltzmann distribution expression of Δ*A* [*E*] ∝ *e*^–(*E* – *E*_f_)/(*k*_B_*T*_c_)^,^10^ where *k*_B_ represents the Boltzmann constant, and *E*_f_ is the quasi-Fermi energy. *T*_c_ can therefore be obtained by fitting the HET of the normalized TA spectrum with the above expression. It should be assigned to the average temperature of electrons with holes as Δ*A* is proportional to the sum of both electron and hole contributions. From the fittings, the initial *T*_c_ at 0.4 ps is about 962 K for control sample, which decreases to 601 K for COF@TCNQ treated sample and 749 K for COF treated sample in the same delay time. Then, the HC cooling lifetime (*t*_cooling_) can be extracted by fitting the curve of *T*_c_ versus delay time using single exponential function, and estimated to be 402 fs, 318 fs, and 367 fs, respectively, for the control, COF@TCNQ-, and COF-doped Spiro-OMeTAD HTL samples (Figure 7g). These results demonstrate smaller HCs distribution in COF@TCNQ and COF treated films because their HCs cooling is faster compared with the control sample. The faster processes of HCs cooling with COF@TCNQ and COF treated samples are consistent with reported cases in literature,^68−70^ which could be due to efficient extraction of the HCs (i.e., hot holes), as shown in Figure 7h (pathway 5). The recovery kinetics of the PB signals also support this conclusion as the PB kinetics were recovered more rapidly in the COF@TCNQ and COF treated samples (Figure S27 and Table S5).

It is necessary to state that the intricate interplay of the HC cooling times can also be affected by pump energy. In general, higher pump fluences (that is, higher excess energies) would result in higher HC temperature at a specific delay time and longer HC lifetime, which yield larger energy loss rates.^8^ This will be unfavorable for achieving highly efficient PCE if the HC extraction is not very efficient in the device. To gain insight of the HC extraction upon excitation with a higher pump fluence, the HC cooling with pump fluences ranging from 5.1 to 40.8 μJ cm^–2^ was further examined. The corresponding TA data measured at higher pump fluences were shown in Figures S28–S33. As expected, *T*_c_ is pump-fluence-dependent and decays slower with increasing pump fluence for all samples, indicating longer HC cooling time at higher pump fluence. However, the magnitude of the increase of the HC cooling times with pump fluence exhibited notable differences for these samples studied here. In Figure 7i, the HC cooling time was plotted as a function of pump fluence, from which we observed that the HC cooling time of the control device exhibits linear dependence on the pump fluence. In contrast, logarithmic dependencies of the HC cooling time on pump fluence were observed in the COF@TCNQ and COF treated samples, suggesting that the HC cooling could be accelerated even at higher pump fluence resulting in longer HC lifetimes. Our observations indicate that the HCs extraction was enhanced by COF@TCNQ and COF. COF@TCNQ results in even more efficient HC extraction due to the increased conductivity.

## Conclusions

In summary, BPTA-TAPD-COF (denotes as COF) and TCNQ-integrated BPTA-TAPD-COF (denotes as COF@TCNQ) were prepared and incorporated into the Spiro-OMeTAD hole transport layer (HTL) of the PSC device. Our study demonstrates that the incorporation of COF and COF@TCNQ acting as multifunctional additives could greatly improve the photovoltaic performance and the stability of perovskite solar cell device by accelerating charge-carrier extraction, suppressing the Li^+^ migration and Li-TFSI agglomeration as well as capturing mobile defects like iodine molecules that may be formed via the oxidation of iodide defects in the perovskite layer. These findings represent an example of using COFs as additives of the Spiro-OMeTAD HTL being able to boost the photovoltaic performance and stability of the PSC devices. Owing to the increased conductivity, the COF@TCNQ led to even more improved photovoltaic performance. Evident from the temperature-dependent PL and femtosecond transient absorption spectroscopic measurements, this large improvement was primarily attributed to the fact that not only the extraction of charge carriers, but also that of hot carriers was enhanced particularly by COF@TCNQ. Thus, the champion PSC device with COF@TCNQ doped Spiro-OMeTAD HTL showed the best power conversion efficiency (PCE) of 24.68% with excellent long-term stability under ambient, thermal, and light soaking conditions. Our work offers a fundamental insight into charge-carrier kinetics at perovskite/HTL interface including dynamics of the hot-carrier cooling, which assists us to understand better the current challenges for effective charge-carrier extraction leading to highly efficient PCE of perovskite solar cells.

## Supporting Information Available

Materials and synthetic procedures; nitrogen adsorption/desorption and pore size distribution; PXRD, HRTEM, and SEM images; solid-state ^13^C CP-MAS spectra; FTIR spectra; *I–V* curves; Tauc plots; AFM morphological images; current density–voltage (*J–V*) curves; water contact angles; UV–vis spectra; UPS spectra; energy level alignment; recovery kinetics; pseudocolor 2D TA data and corresponding normalized TA spectra; and plots of hot-carrier temperature versus delay time (PDF)
