# Supplementary material for: Engineering the Hole Transport Layer with a Conductive Donor–Acceptor Covalent Organic Framework for Stable and Efficient Perovskite Solar Cells
Source: ACS Cent Sci. 2024 Jun 14;10(7):1383–95. doi: 10.1021/acscentsci.4c00416 (PMC11273455; doi:10.1021/acscentsci.4c00416)
Supplement: Supplementary file 1 — oc4c00416_si_001.pdf [file oc4c00416_si_001.pdf]

Supporting Information

Engineering the Hole Transport Layer with a Conductive Donor-Acceptor Covalent Organic Framework for Stable and Efficient Perovskite Solar Cells

Shihuai Wang,[†,‡,§] Tai Wu,[†,§] Jingjing Guo,[‡] Rongjun Zhao,[†] Yong Hua,[†]* Yanli Zhao[‡]*

[†]Yunnan Key Laboratory for Micro/Nano Materials & Technology, School of Materials and Energy, Yunnan University, Kunming 650091, Yunnan, China

[‡]School of Chemistry, Chemical Engineering and Biotechnology, Nanyang Technological University, Singapore 637371, Singapore

[§]These authors contributed equally to this work.

*Email: huayong@ynu.edu.cn; zhaoyanli@ntu.edu.sg.

Experimental section

Materials

All solvents and reagents for the synthesis and device fabrication, if not stated otherwise, were purchased from commercial sources and used without further purification. 2,5-Bis(2-propynyloxy)terephthalaldehyde (BPTA), N,N,N',N'-tetrakis(4-aminophenyl)-1,4-phenylenediamine (TAPD), and 7,7',8,8'-tetracyanoquinodimethane (TCNQ) were purchased from BLD Pharmatech Ltd. Acetic acid (≥99%), benzyl alcohol (anhydrous, ≥99.8%), chlorobenzene (CB, ≥99.5%), N,N-dimethylformamide (DMF, ≥99%), dimethyl sulfoxide (DMSO, 99%), isopropanol (IPA, ≥99%) and mesitylene (≥99.5%) were purchased from Sigma-Aldrich chemicals. Lead iodide (PbI_2, ≥98.0%) was purchased from TCI. Formamidinium iodide (FAI, ≥99.9%). tert-Butylpyridine (*t*-BP), lithium bis(trifluoromethanesulfonyl)imide (Li-TFSI), methylammonium bromide (MABr, ≥99.9%), methylammonium Chloride (MACl, ≥99.9%), and Sprio-OMeTAD (2,2,7,7-tetrakis(N,N-di-p-methoxyphenylamine)-9,9'-spirobifluorene) were purchased from Advanced Election Technology Co., Ltd. SnO_2 colloid precursor (tin (IV) oxide, 15% in H_2O colloidal dispersion) was purchased from Alfa Aesar.

Synthesis

Synthesis of BPTA-TAPD COF. In a 10 mL Pyrex tube, a solid mixture of TAPD (12.3 mg, 0.026 mmol) with BPTA (12.6 mg, 0.052 mmol) was suspended in mesitylene and benzyl alcohol (2 mL, 1/1.5 in v/v). Subsequently, the suspension was sonicated for 2 min, followed by adding acetic acid (6 M, 100 μL). Then, the Pyrex tube was degassed by three freeze-pump-thaw cycles, sealed off by flame and heated at 100 °C in an oven for 72 h. The resulting red-brown precipitate was filtrated and washed with acetone and THF for 3 times, and then subjected to Soxhlet extraction with the mixture of THF/MeOH as the solvent for 24 h to further remove unreacted monomers and oligomers. The resulting dark red microcrystalline powder was dried at 100 °C under vacuum overnight to give the pure COF with which a yield of 85% was obtained.

Synthesis of BPTA-TAPD COF@TCNQ. The synthesis of covalent TCNQ immobilization in COF was done according to reported procedure in literature.[S1] In a 10 mL Schlenk flask, a solid mixture of BPTA-TAPD COFs (20 mg) and TCNQ (40.8 mg, 0.2 mmol) was dispersed in chloroform (2 mL). The reaction mixture was then degassed through three freeze-pump-thaw cycles, and the

suspension was stirred at 50 °C for 72 h. The dark precipitate was obtained and filtered, followed by washing procedure with MeOH, chloroform, THF and ethyl acetate. Finally, the precipitate was subjected to Soxhlet extraction with THF as the solvent for 48 h to further remove the physisorbed TCNQ in the COF. A control experiment was conducted by mixing BPTA-TAPD COFs (20 mg) with TCNQ (40.8 mg, 0.2 mmol) in chloroform (2 mL), and this mixture stands at room temperature for 24 h. The covalent TCNQ immobilization in the COF was finally characterized by solid-state NMR and FT-IR spectroscopies.

Device fabrication

Before preparation of the film, the conductive indium tin oxide (ITO) substrate was cleaned by sequentially sonicating with detergent, deionized water, acetone and anhydrous ethanol (each for 20 min). The cleaned and dried ITO substrate was further treated with ultraviolet ozone for 20 min. Then, the SnO_2 colloidal precursor (40 ml, 2.67%, diluted by milli-Q water) was deposited onto the substrate via spin-coating process at 4,000 rpm for 30 s and further annealed in air at 150 °C for 30 min to obtain the electron transport layer (ETL). Before taking into N_2-filled glovebox to fabricate perovskite film, the SnO_2-coated ITO substrate needs to be soaked under ultraviolet ozone for 20 min to improve the surface wetting. The perovskite layer was prepared via a two-step method. First, the PbI_2 solution (1.5 M dissolved in a mixture of DMF:DMSO (9:1 in v/v)) was spin-coated onto SnO_2-coated ITO substrate at 1,500 rpm for 30 s followed by annealing at 70 °C for 1 min, and then cooled to room temperature. Then, a solution of FAI: MACl: MABr (90 mg: 9 mg: 9 mg in 1ml IPA) was spin-coated onto the PbI_2 layer at a speed of 1,800 rpm for 30 s. Subsequently, the perovskite precursor film was taken out from the glove box to ambient air with a relative humidity of 30-40% for thermal annealing at 150 °C for 15 min. It is important to note that the humidity is very critical for obtaining high crystallinity of the perovskite film and high performance of the device. This could be explained that the perovskite precursor could absorb water onto the surface, which could promote the perovskite crystal growth.[S2] After perovskite formation, the samples were again transferred into N_2-filled glove box for deposition processing of hole transport layer (HTL). The HTL was deposited on top of the perovskite layer at a speed of 3,000 rpm for 30 s. Three kinds of HTL precursors including the control, COF@TCNQ- and COF-doped HTL solutions were used. Before use, the COF@TCNQ and COF were processed by ball milling method to form colloidal and homogeneous solutions. For the control HTL deposition, a precursor solution

consisted of Spiro-OMeTAD (90 mg), *t*-BP (37 μL), and Li-TFSI (22 μL, 520 mg mL^{-1} in acetonitrile) in CB (1 mL). For preparation of COF@TCNQ-doped HTL precursor solution, COF@TCNQ solution (7 mL, 2 mg/mL in acetonitrile) as optimal condition was added to the solution used for the control HTL deposition. COF-doped HTL precursor solution was prepared by mixing COF solution (5 mL, 2 mg/mL in acetonitrile) with the solution used for the control HTL deposition. After oxidation overnight, gold film (80 nm) was deposited via thermal evaporation under a vacuum of 2×10^{-4} Torr. A non-refractive mask was used to determine the effective area of 0.0625 cm^2.

Methods and Characterizations

Structural simulation. Molecular modelling and Pawley refinement were conducted using Reflex, a software package for crystal determination using Powder XRD pattern, implemented in BIOVIA Materials Studio modelling version, 2019 (Dassault System). For structural determination of COFs, the lattice model was optimized using the Materials Studio Forcite molecular dynamics module. Then, Pawley refinement was performed to optimize the lattice parameters. Pseudo-Voigt profile function was used for the profile fitting (peak broadening, peak asymmetry, and zero shift error were under consideration) until obtaining the optimized lattice parameters with the converged Rwp value.

Computational studies. COF-fragment and COF@TCNQ-fragment were optimized using the density function theory (DFT) and time-dependent density functional theory (TD-DFT) method, respectively, with M06-2X hybrid functional at the basis set level of 6-311G(d,p).

Fourier transform infrared (FT-IR) spectroscopy. FT-IR spectra were carried out on a Perkin-Elmer 1760 FT-IR spectrometer, and the samples for FT-IR data collection were prepared as KBr pellets.

Powder X-ray diffraction (PXRD). PXRD patterns were acquired on a Bruker D8 Advance diffractometer in reflection geometry using Cu-Kα radiation (λ = 1.54178 Å) operated at 40 kV and 40 mA. The data were collected from 2 to 30 2θ degrees with a step size of 0.02 degrees. The samples were prepared as a thin layer by spreading as-synthesized powders onto the circular recess of a silicon sample holder.

Solid-state nuclear magnetic resonance (NMR) spectroscopy. ^{13}C solid-state cross polarization magic-angle spinning (CP-MAS) NMR spectra were measured on a Bruker Avance III HD 600 MHz (14.1 T) wide-bore NMR spectrometer equipped with a 4 mm HX MAS probe. Samples were

mounted tightly into a 4 mm zirconium oxide rotor. The data were collected with 13 kHz MAS spinning rate, 2 ms CP contact time and 3 s recycle delay. ^{7}Li NMR spectra were measured with direct polarization, a spectral width of 138.889 kHz, relaxation time of 5 s and a $\pi/2$ pulse (66 kHz).

Nitrogen (N_2) adsorption-desorption isotherms. N_2 sorption isotherms were measured using a Quantachrome Instrument Autosorb-iQ (Boynton Beach, Florida USA) at 77 K within pressure ranges of P/P_0 = 0.001 to 0.98. Prior to the measurements, the samples (~ 15 mg) were activated and degassed at 100 ºC overnight. The Brunauer-Emmett-Teller (BET) surface areas were calculated from the resulting isotherms, and the pore size distribution was calculated based on the N_2 sorption isotherm by using the QSDFT absorption model (a carbon model containing kernel for cylindrical pores).

Scanning electron microscopy (SEM). SEM images were recorded with an a JEOL JSM-7600F microscope equipped with a field emission gun operated at 5 kV. Prior to the measurements, the samples were prepared by depositing diluted suspension on silicon wafers, followed by air drying and sputtering with Pt.

High-resolution transmission electron microscopy (HR-TEM). HR-TEM images were recorded on a JEOL JEM 2010UHR electron microscope operated at 200 kV and equipped with Gatan CCD camera for high resolution imaging of materials. The samples were prepared by placing a droplet of the sample suspension onto a TEM carbon-coated copper grid, followed by air drying.

Atomic force microscopy (AFM). The surface microstructure and roughness of COF@TCNQ or COF doped Spiro-OMeTAD/perovskite films were investigated by AFM (CSPM 5500, Being Nano-Instruments).

Solid-state diffuse reflectance Ultraviolet–visible (DR-UV-Vis) spectroscopy. Solid-state DR-UV-Vis spectra were recorded on a Shimadzu UV-2600 spectrometer equipped with an ISR-2600Plus integrating sphere attachment.

General procedure for iodine vapor sorption. In typical static sorption of iodine, the dried COF sample (25 mg) was added in two open small vials (2 mL), respectively. Then, this COF-loaded small vail was transferred into a large glass vial (20 mL) containing iodine (1 g). The large vial was subsequently sealed and kept in an oven at 65 °C. After a certain period of adsorption time, the large vial was cooled to room temperature and the COF-loaded small vial was taken out. The small vial was weighed and then placed back into the large vial. The large vial was sealed again and put

back in the oven at 65 °C to continue iodine adsorption until the COF-loaded small vial reached saturation.

General procedure for Li-TFSI adsorption. Typically, the dried COF sample (50 mg) was immersed in acetonitrile solution of Li-TFSI (1 M) for 24 h. After that, the suspension was filtered, and then the filtered COF sample was washed thoroughly with acetonitrile and THF to remove free Li-TFSI. The sample was dried under vacuum at 50 °C for 48 h, and then sent for ^{7}Li NMR spectra measurements.

General characterizations of the devices. Photocurrent density-voltage (*J-V*) characteristic curves were measured under 100 mW/cm^2 (AM 1.5G illumination) using a Newport solar simulator (model 91160) and a Keithley 2400 source/meter. A certified reference solar cell (Fraunhofer ISE) was used to calibrate the light source for an intensity of 100 mW/cm^2. Incident photon-to-current conversion efficiency (IPCE) spectra were recorded using a computer-controlled setup consisting of a Xenon light source (Spectral Products ASB-XE-175), a monochromator (Spectra Products CM110), and a potentiostat (LabJack U6 DAQ board), calibrated by a certified reference solar cell (Fraunhofer ISE). The voltage scan rate was 10 mV·s^{-1} and no device preconditioning, such as light soaking or forward voltage bias applied for long time, was applied before starting the measurement. The cells were masked with a black metal mask, which leaves the active area to 0.0625 cm^2 and reduces the influence of the scattered light.

Conductivity tests. A two-probe method was used to determine the conductance σ (S/cm) by mean of following expression, $\sigma = \frac{Id}{VA}$,[S3] where *I* is the current, *d* is the thickness of the sample, *V* is the voltage and A is the active area of the sample. Thus, σ can be estimated by fitting a linear *I-V* curve measured using a Keithley 2400 semiconductor characterization system. To measure the conductivity of COF powders, the COF sample was placed into a pressing die and pressed under high pressure to prepare a kind of pellet with the diameter of ~0.6 cm and the thickness of ca. 2 mm. Two pieces of gold wires were connected to both sides of the COF pellet. The *I-V* curve measurement was conducted in the voltage sweeping from -1.0 V to +1.0 V. To measure the electrical conductivity of the HTL, the device with a structure of ITO/HTL without and with COF@TCNQ or COF/Au was fabricated, and the conductivities of these films were measured by the method used for that of COF pellets.

Hole mobility measurements. The space charge limited currents (SCLCs) method was employed to estimate the hole mobility. For this measurement, the device with a configuration of ITO/PEDOT:

PSS/HTL without and with COF@TCNQ or COF/Au was fabricated. The PEDOT: PSS layer (about 40 nm of thickness) was spin-coated on the ITO substrates at 4,000 rpm for 30 s, followed by thermal annealing at 170 °C for 20 min in the ambient air environment. The substrates were then transferred into an N_2-filled glovebox for further fabrication steps. Other functional layers were prepared according to the above device fabrication process. In glove box, the HTL solutions without and with COF or COF@TCNQ was spin-coated at 4000 rpm to fabricate corresponding films. The amount of each component in the HTL solution was presented in the device fabrication section. Finally, 80 nm of gold was thermally evaporated onto the active layer under high vacuum (less than 10-6 mbar). The current-voltage (*I-V*) curves of these hole-only devices were measured with measured with a Keithley 2400 Source meter under dark conditions. The hole mobility (m_e) was estimated by fitting the dark J-V curve according to the Mott-Gurney equation, $J = \frac{9}{8}\mu_e\varepsilon_0\varepsilon_r\frac{V^2}{d^3}$,[S4] where J is the current density, ε_o is the vacuum permittivity (8.85×10^{-12} F/m or 8.85×10^{-14} F/cm), ε_r is the relative permittivity of the material (thus, the dielectric constant ($\varepsilon_r/\varepsilon_o$) is normally taken to approach 3 for organic semiconductors),[S5] V is the applied bias, and d is the film thickness.

Electrochemical characterizations. Electrochemical experiments including transient photovoltage (TPV) decays, electrochemical impedance spectroscopy and light-dependent open-circuit voltage (V_{OC}) were performed with the ZAHNER (PP211) electrochemical workstation. V_{OC} was measured with various intensities of light (I) spanning from 10 to 100 mW·cm^{-2}. When plotting V_{OC} as function of I, V_{OC} was found to vary linearly with $\ln(I)$, as described by the following equation, $V_{OC} = \frac{n_{id}KT}{e}\ln(I) + constant$,[S6] where e is the elementary charge, K is the Boltzmann constant, T is the temperature, I is the incident light intensity, and n_{id} is diode ideality factor. The values of n_{id} for the control, COF@TCNQ- and COF-treated samples PSC devices, determined from the slopes of V_{OC} as function of I, are found to be 1.52, 1.35 and 1.47, respectively.

Stead-state photoluminescence (PL) and time-resolved PL spectroscopies. The steady-state PL spectra and the time-resolved decays were measured using Edinburgh FLS1000 (UK) exciting at 475 nm. The temperature-dependent PL spectra were measured using this spectrometer equipped with a temperature-controlled sample holder. Briefly, the samples were placed into the sample chamber, in which the temperature was controlled using liquid nitrogen. Then, the PL spectra were collected within temperature ranges of 77 K to 273 K.

Femtosecond transient absorption (fs-TA) spectroscopy. The fs-TA measurements were carried out using optical pump-probe spectroscopy. Briefly, an 800 nm fundamental output generated with a mode-locked Ti-sapphire laser amplifier (Spectra-Physics, ~100 fs FWHM, 2 KHz) was split to generate pump and probe pulses. The pump beam (75%) was passed through an optical parametric amplifier (TOPAS) to obtain the 400 nm excitation light. The probe pulse (white light) was produced by focusing the other 25% beam on a CaF_2 crystal. Instrument response is about 150 fs. The film samples were continuously translated to avoid heating and permanent degradation. The pump power (400 nm) at the sample was adjusted as requested.

Supporting Figures and Tables

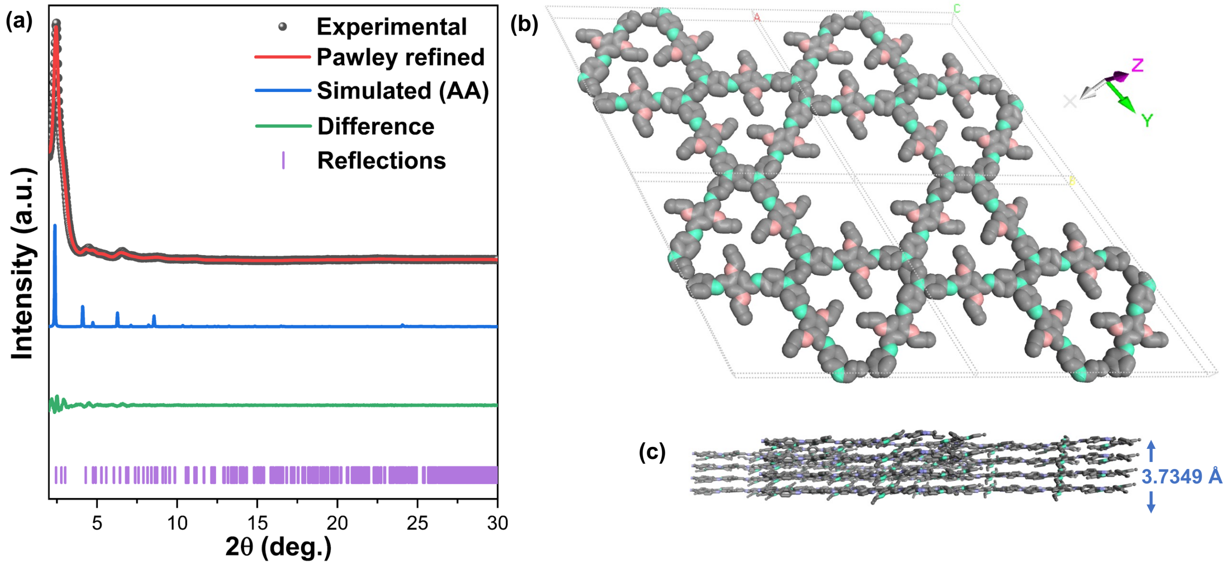

Figure S1. (a) Experimental, Pawley refined, simulated PXRD patterns, the refinement difference and the reflected points of BPTA-TAPD COF. (b) Top and (c) side views of structural simulation for BPTA-TAPD COF; H atoms are omitted for clarity.

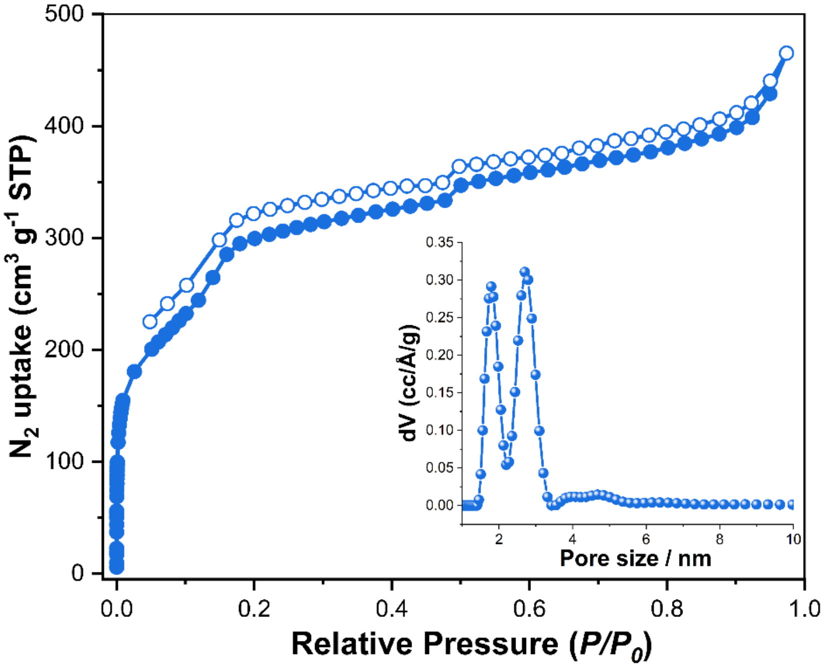

Figure S2. N_2 sorption isotherms at 77 K and the pore size distribution (inset) of BPTA-TAPD COF (parent COF). The Brunauer–Emmett–Teller (BET) area was calculated to be 976 m^2/g.

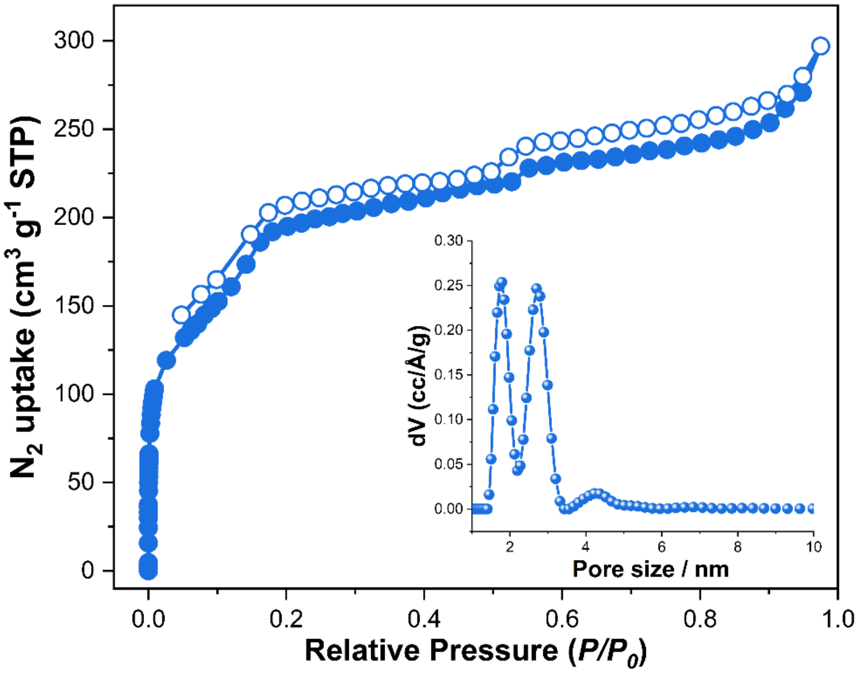

Figure S3. N_2 sorption isotherms at 77 K and the pore size distribution (inset) of BPTA-TAPD COF@TCNQ (COF@TCNQ). The Brunauer–Emmett–Teller (BET) area was calculated to be 664 m^2/g.

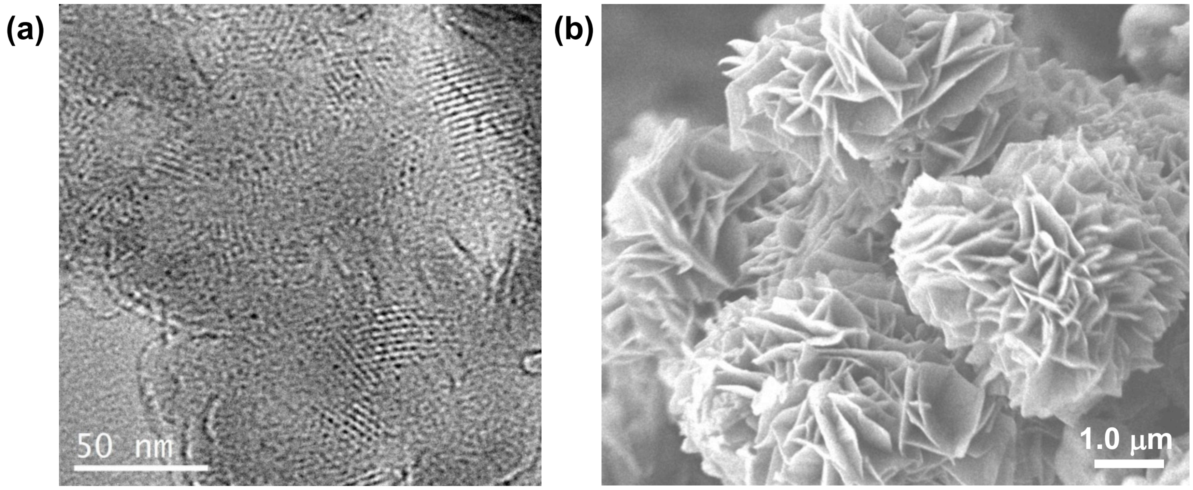

Figure S4. (a) HRTEM image showing a lattice spacing (010) of about 2.4 nm and (b) SEM image indicating rosebud-like spherical morphology for BPTA-TAPD COF (parent COF).

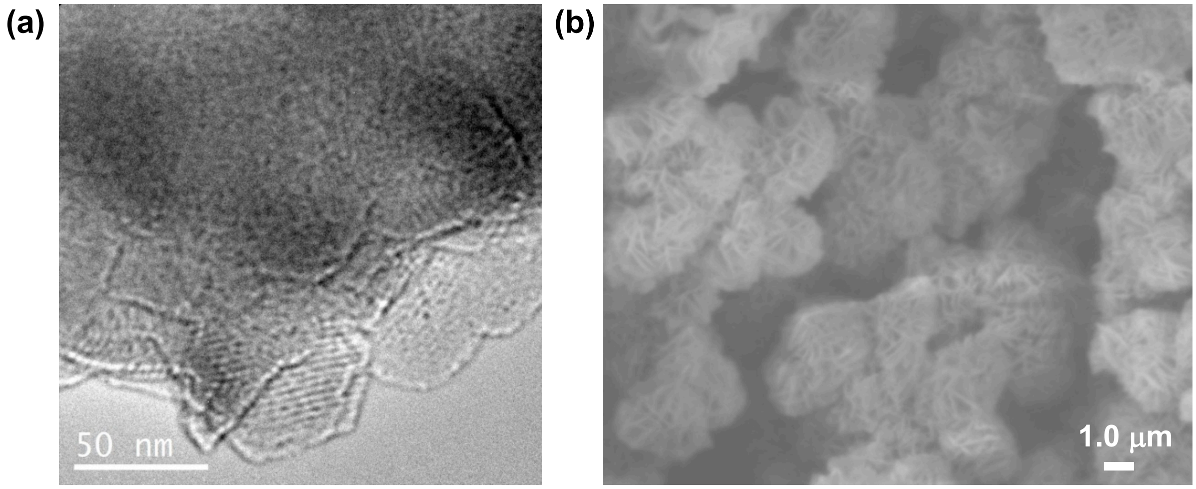

Figure S5. (a) HRTEM image showing a lattice spacing (010) and (b) SEM image indicating rosebud-like spherical morphology for BPTA-TAPD COF@TCNQ (COF@TCNQ).

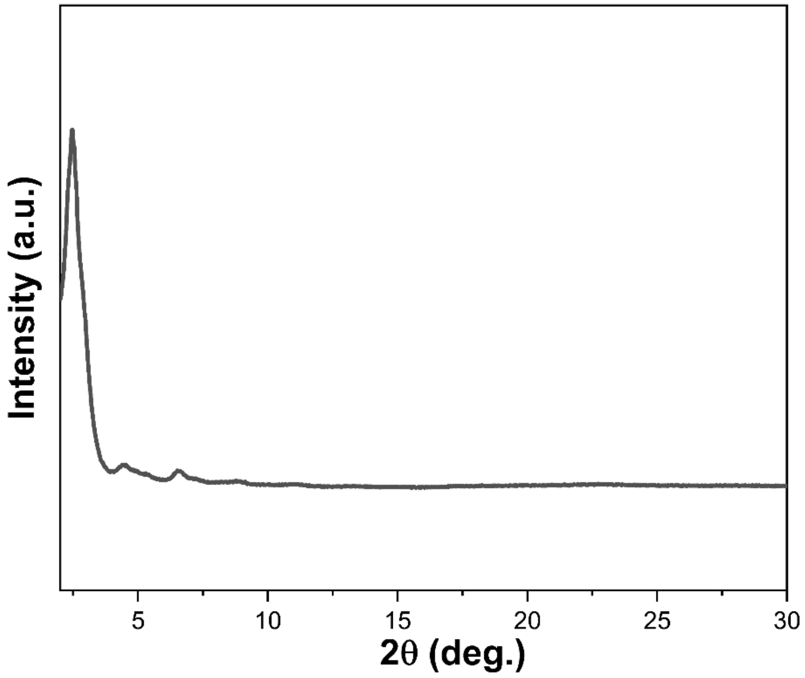

Figure S6. Experimental PXRD pattern of BPTA-TAPD COF@TCNQ.

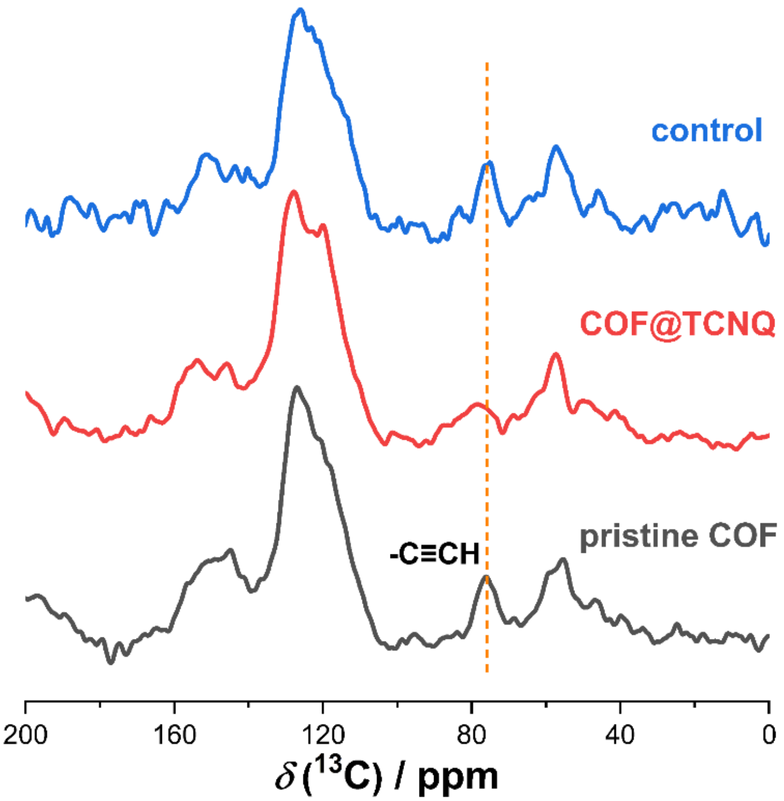

Figure S7. Solid-state ^{13}C CP-MAS spectra of parent COF (black), COF@TCNQ (red) and control (blue) corresponding to the structures. The control experiment was conducted by simply mixing the COF with TCNQ in chloroform at room temperature for 24 h, followed by washing of Soxhlet extraction with THF as the solvent.

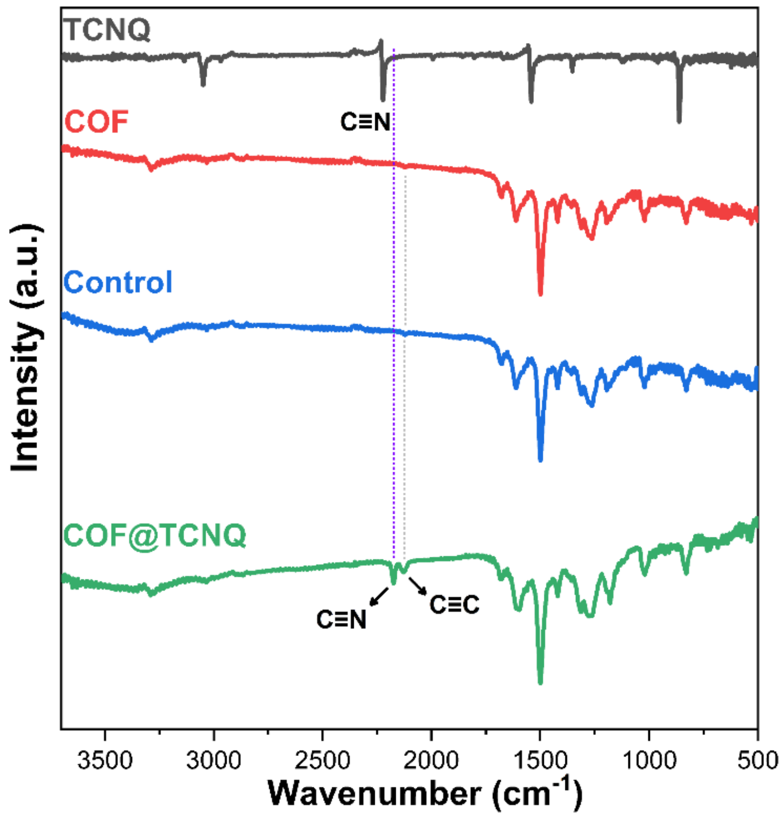

Figure S8. FT-IR spectra of TCNQ (black), parent COF (red), control (blue) and COF@TCNQ (green). The control experiment was conducted by simply mixing the COF with TCNQ in chloroform at room temperature for 24 hours, followed by washing of Soxhlet extraction with THF as the solvent.

Table S1. Fitted parameters of the PL decays for COF and COF@TCNQ powders.

	**A_1**	**t_1 /ps**	**A_2**	**t_2 /ps**	**t_{ave} /ps**
**COF**	10.05	499.48	89.47	499.49	499.49
**COF@TCNQ**	198.35	402.12	52.92	402.12	402.12

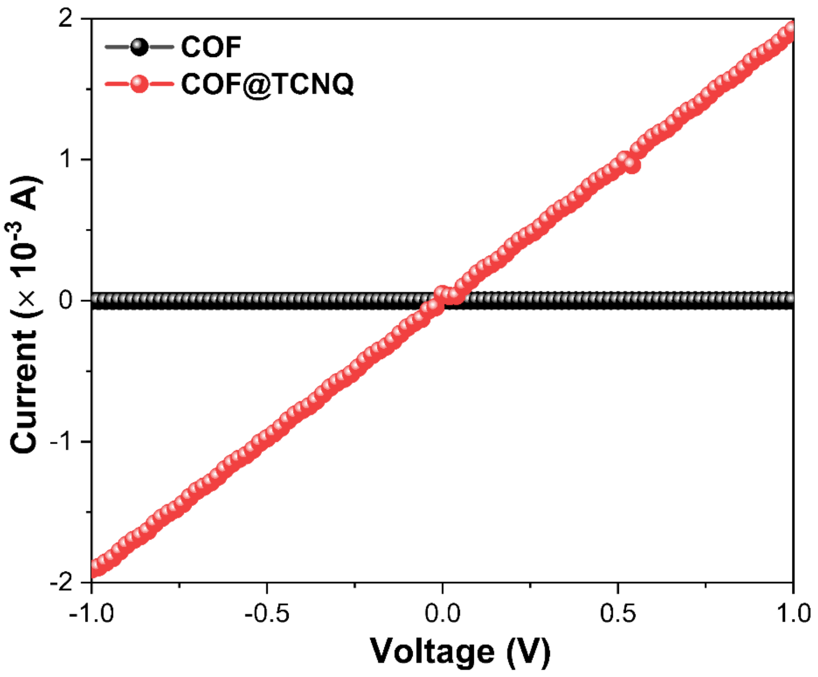

Figure S9. *I-V* curves of parent COF and COF@TCNQ pellets measuring their conductivities.

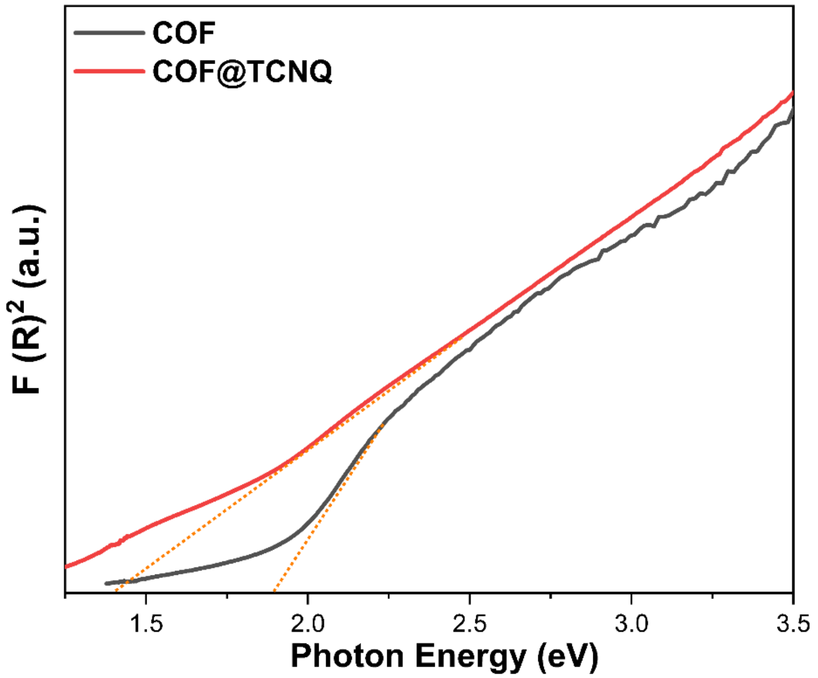

Figure S10. Tauc plots of parent COF and COF@TCNQ powders originated from their diffuse reflectance UV-Vis spectra, which determine the bandgaps.

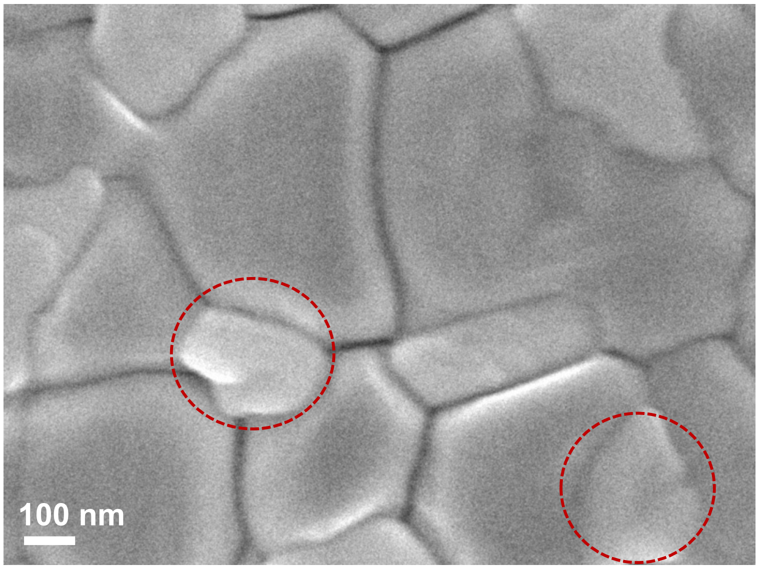

Figure S11. SEM image of perovskite layer. The dashed red circles show the presence of the uncoordinated defects.

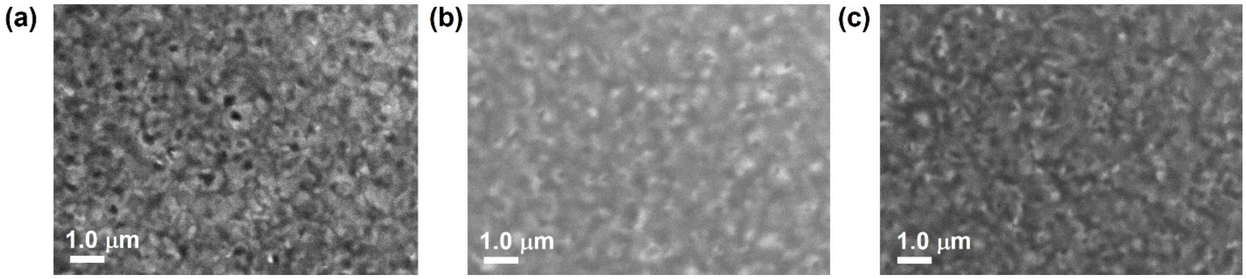

Figure S12. SEM morphological images of (a) the freshly prepared perovskite/Spiro-OMeTAD, (b) perovskite/Spiro-OMeTAD+COF@TCNQ, and (c) perovskite/Spiro-OMeTAD+COF films.

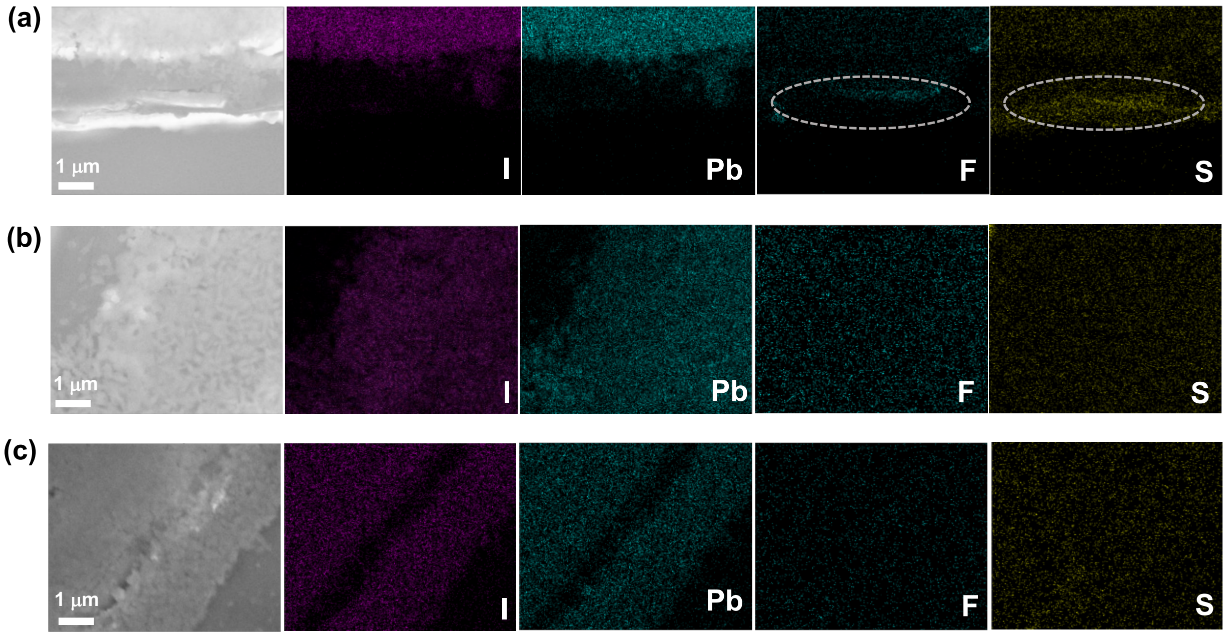

Figure S13. SEM morphological images and EDX analysis of (a) perovskite/Spiro-OMeTAD, (b) perovskite/Spiro-OMeTAD+COF@TCNQ, and (c) perovskite/Spiro-OMeTAD+COF films after stored at ambient conditions for 35 days.

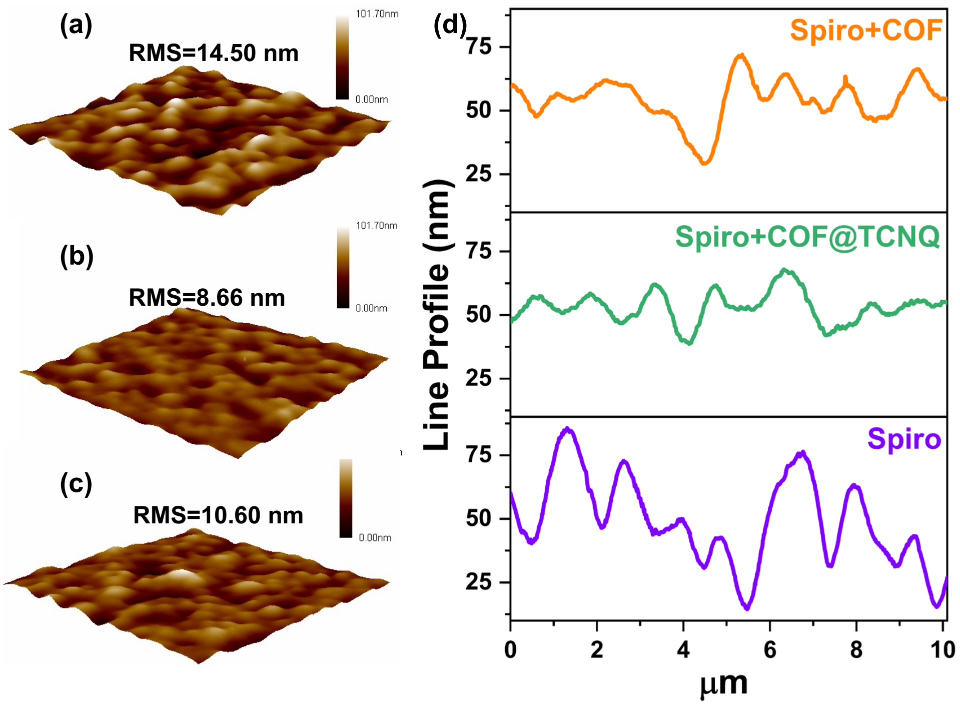

Figure S14. AFM morphological images of (a) perovskite/Spiro-OMeTAD, (b) perovskite/Spiro-OMeTAD+COF@TCNQ and (c) perovskite/Spiro-OMeTAD+COF films. (d) Line profiles of the AFM images for the corresponding films.

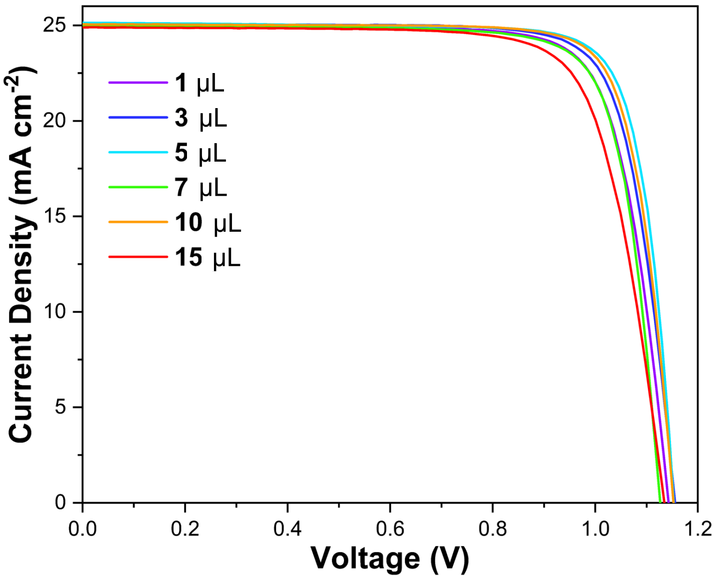

Figure S15. Current density-voltage (*J-V*) curves of perovskite devices based on the Spiro-OMeTAD layer with treatment of various volumes of the parent COF (2 mg/mL).

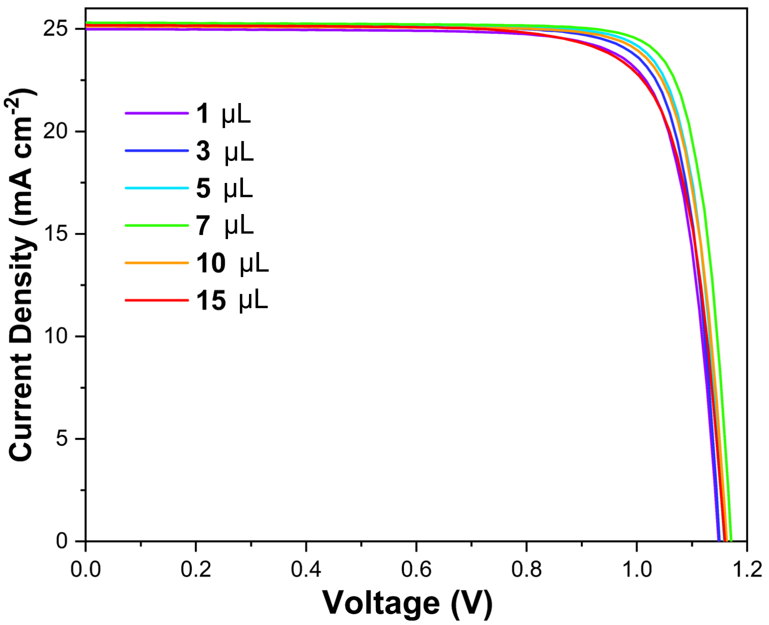

Figure S16. Current density-voltage (*J-V*) curves of perovskite devices based on the Spiro-OMeTAD layer with treatment of various volumes of the COF@TCNQ (2 mg/mL).

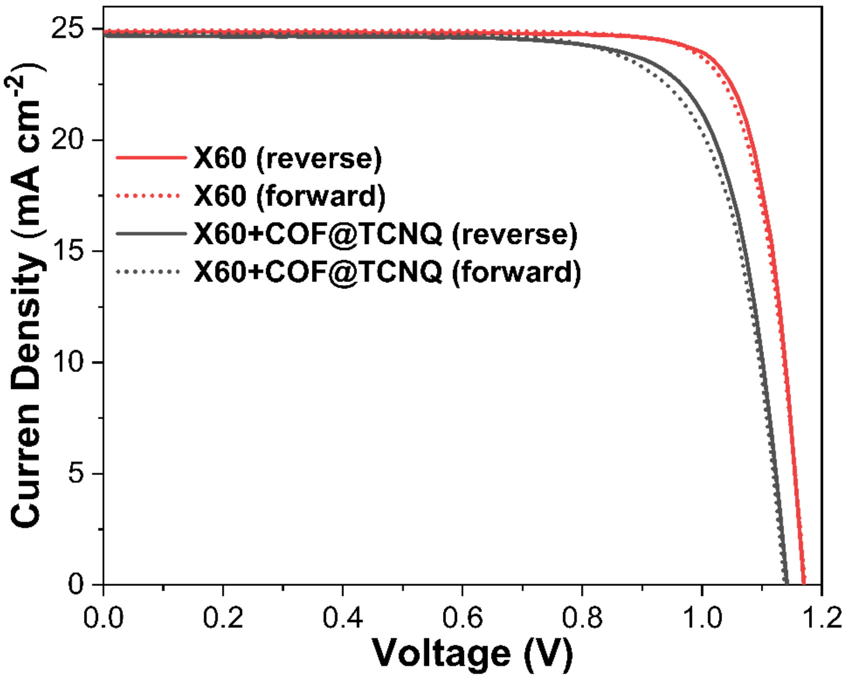

Figure S17. *J-V* curves of the PSC devices with X60 (control) and X60+COF@TCNQ (target) HTLs measured under reverse scanning (solid lines) and forward scanning (dashed lines) modes.

Table S2. Photovoltaic parameters of the devices based on X60 HTM. HI indicates the hysteresis of the devices under reverse scanning and forward scanning.

Cell	Scan mode	V_{OC} /V	J_{SC} /mA cm^{-2}	*FF* /%	PCE /%	HI /%
**X60**	reverse	1.142	24.72	77.56	21.89	3.2
	forward	1.138	24.78	75.19	21.20	
**X60+COF@TCNQ**	reverse	1.171	24.87	82.51	24.03	1.5
	forward	1.170	24.91	81.27	23.68	

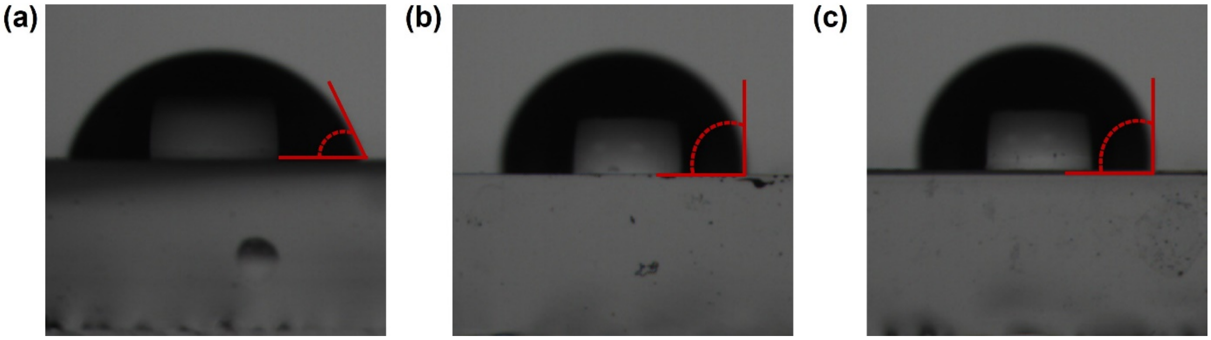

Figure S18. Water contact angles of (a) the control, (b) COF@TCNQ-treated, and (c) COF-treated perovskite thin films.

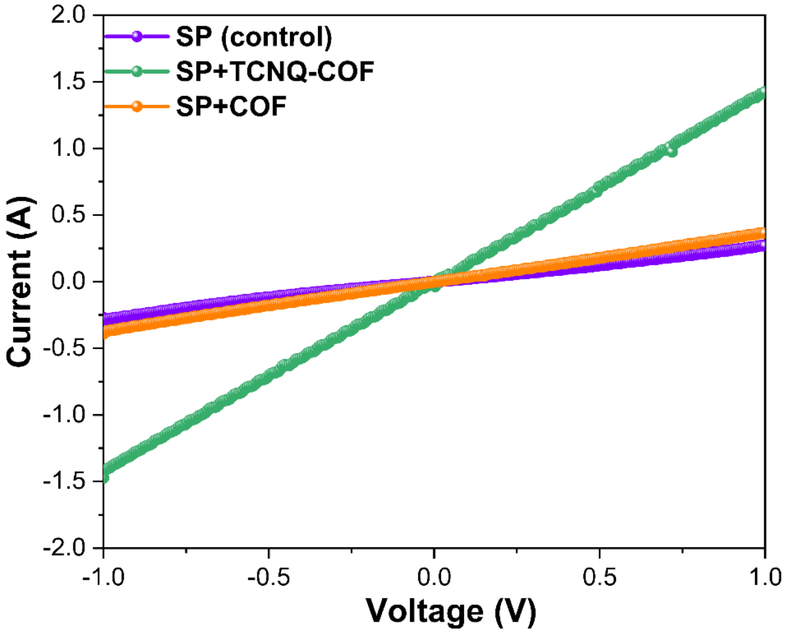

Figure S19. *I-V* curves of the control, COF@TCNQ- and COF-treated films measuring their conductivities.

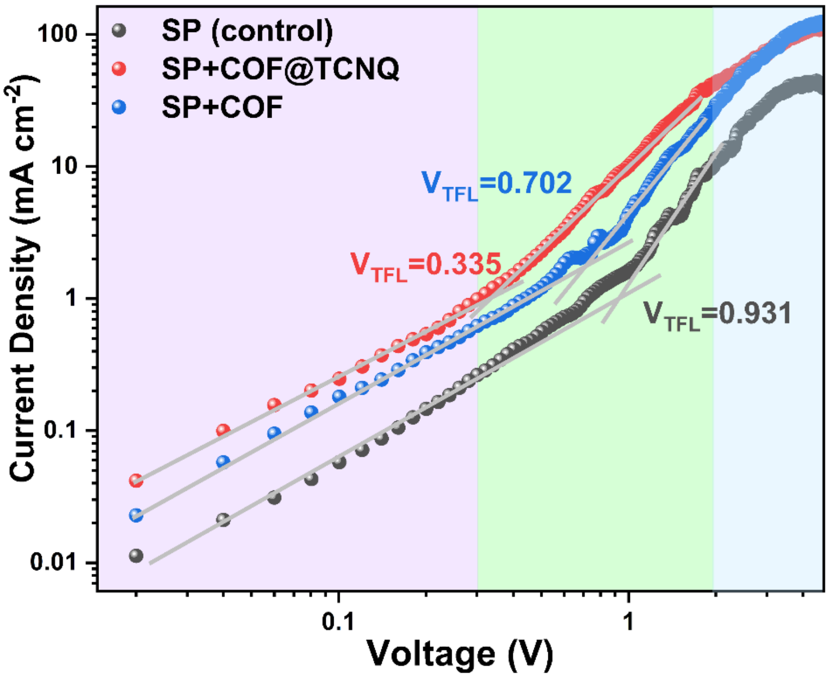

Figure S20. *J−V* curves of hole-only devices in the dark (device configuration: ITO/PEDOT:PSS/perovskite/HTL/Au); HTL refers to Spiro-OMeTAD or COF@TCNQ treated Spiro-OMeTAD or COF treated Spiro-OMeTAD.

Table S3. Calculation parameters of hole trap-state density (N_t) for the control, COF@TCNQ- and COF-treated devices. SP represents the Spiro-OMeTAD, applying this definition thereafter.

	**Onset voltage (V_{TFL}) / V**	**Thickness (*L*) / nm**	**Dielectric constant (e/e_0)**	**N_t /cm^3**
**SP (control)**	0.931	150	3	1.95×10^{16}
**SP+COF@TCNQ**	0.335	150	3	7.00×10^{15}
**SP+COF**	0.702	150	3	1.47×10^{16}

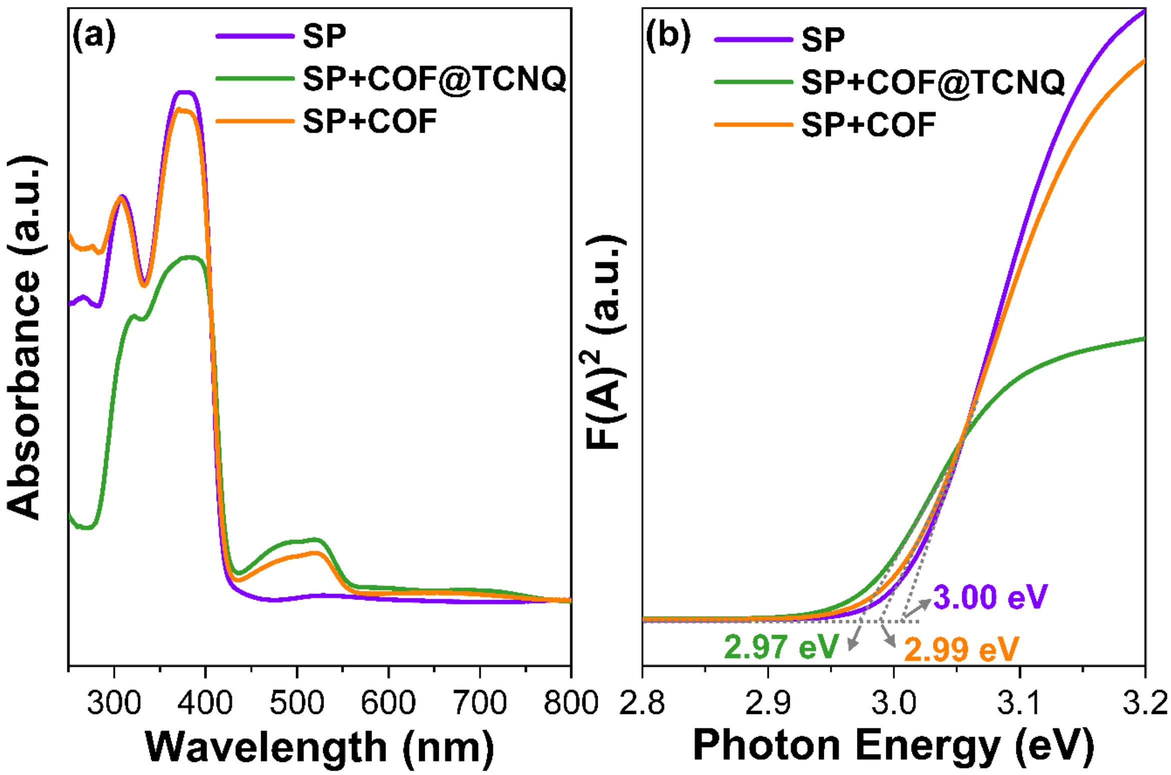

Figure S21. (a) UV–vis absorption spectra and (b) Tauc plots of HTLs films.

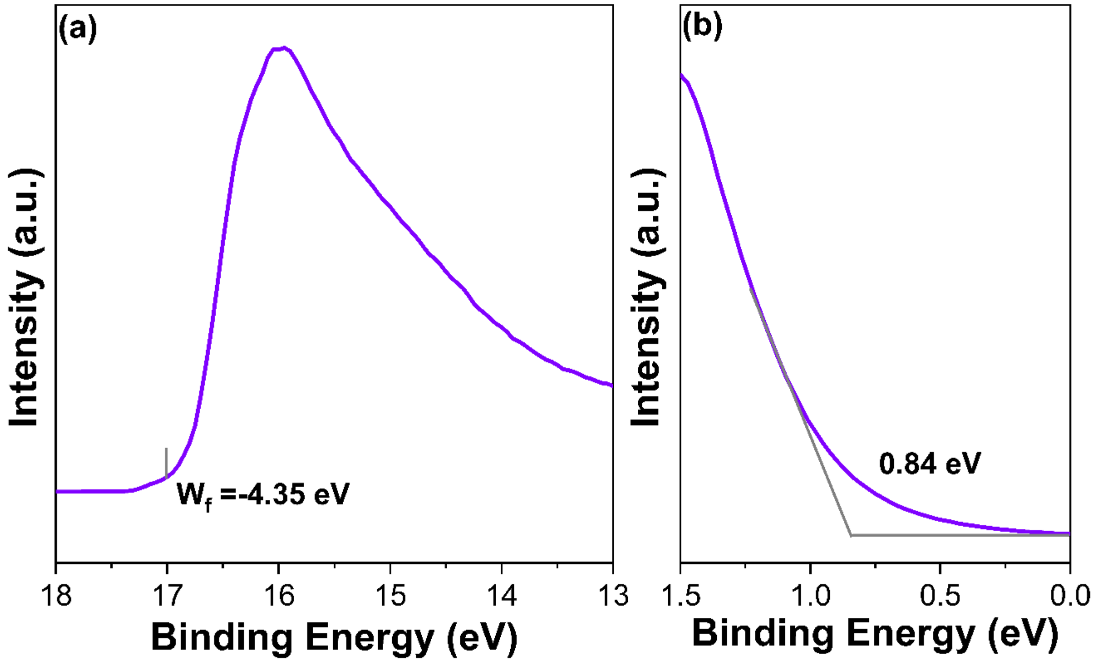

Figure S22. UPS spectra of Spiro-OMeTAD HTL film. (a) Cut-off region determining the work function (W_f) and (b) onset region potential (E_{onset}).

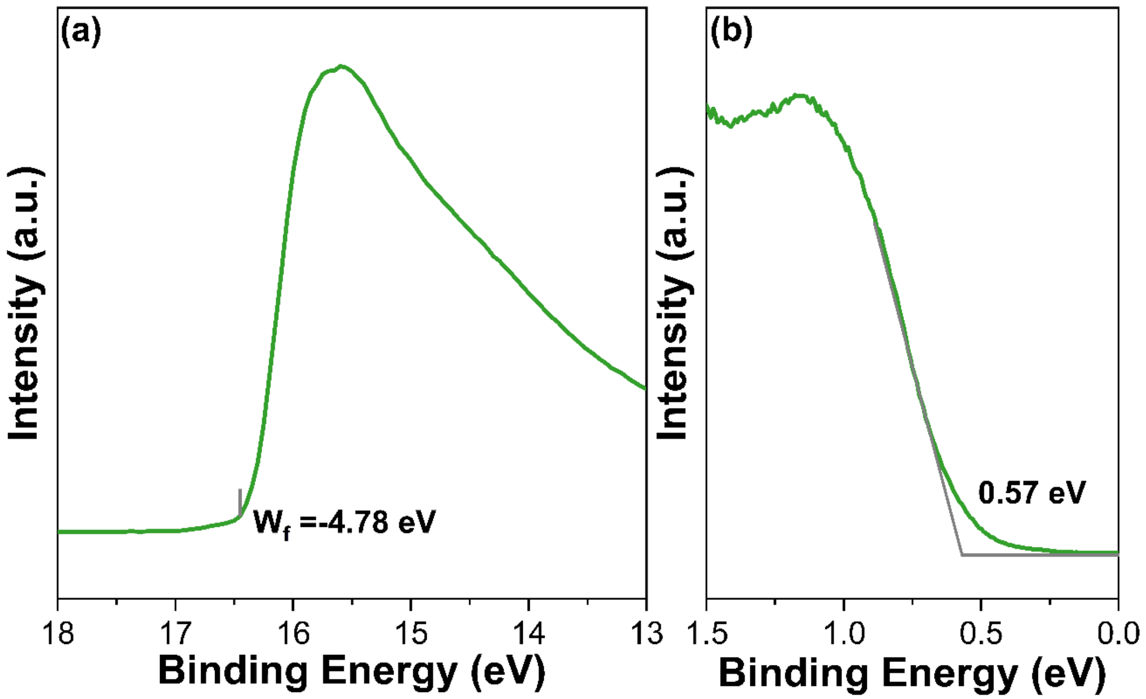

Figure S23. UPS spectra of Spiro-OMeTAD+COF@TCNQ HTL film. (a) Cut-off region determining the work function (W_f) and (b) onset region potential (E_{onset}).

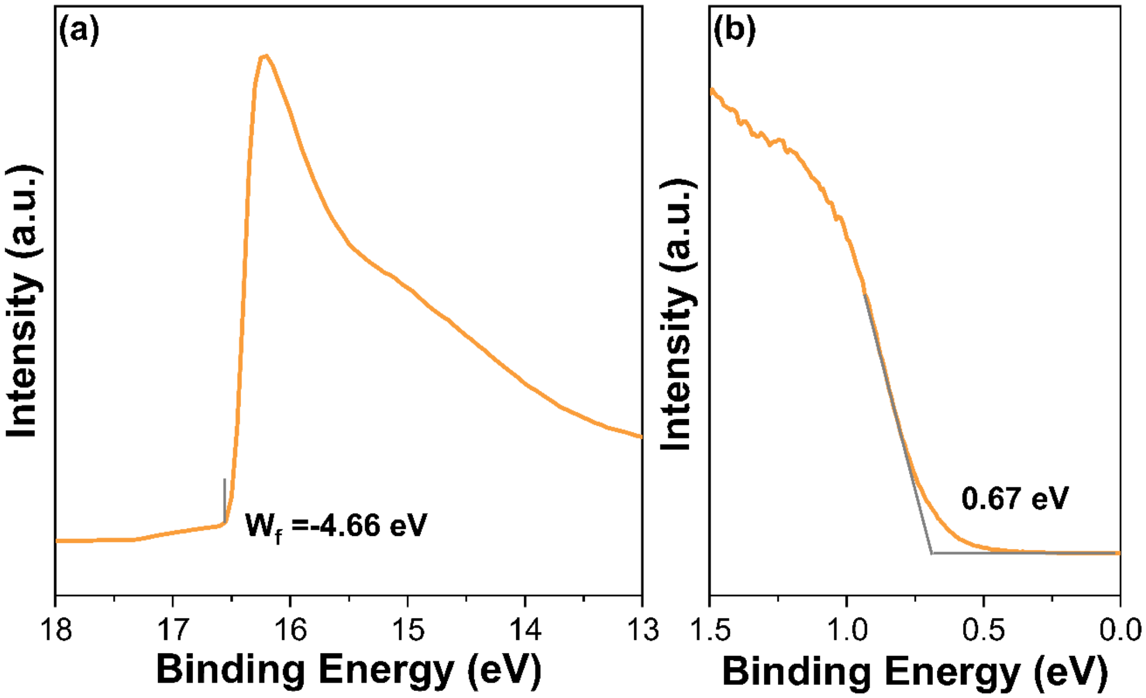

Figure S24. UPS spectra of Spiro-OMeTAD+COF HTL film. (a) Cut-off region determining the work function (W_f) and (b) onset region potential (E_{onset}).

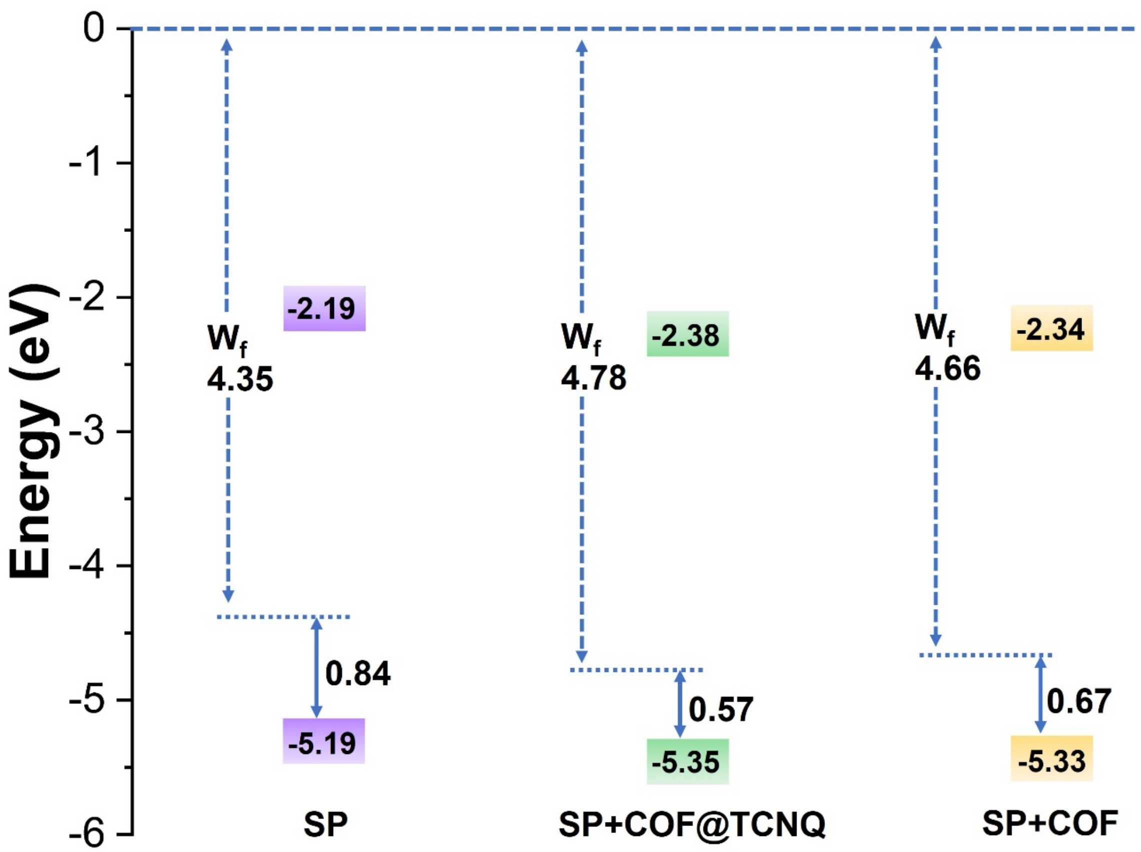

Figure S25. Energy level alignment for the control, COF@TCNQ- and COF-doped Spiro-OMeTAD HTL films.

Table S4. Fitted parameters of the time-resolved photoluminescence decays for the perovskite films without and with HTL.

	**A_1**	**t_1 /ns**	**A_2**	**t_2 /ns**	**t_{ave}**
**PVSK**	135.82	2238.38	109.94	2236.76	2237.66
**PVSK/SP**	198.76	37.93	83.02	551.11	189.13
**PVSK/SP+COF@TCNQ**	69.88	10.04	46.83	254.11	107.93
**PVSK/SP+COF**	92.14	35.16	39.46	462.24	163.22

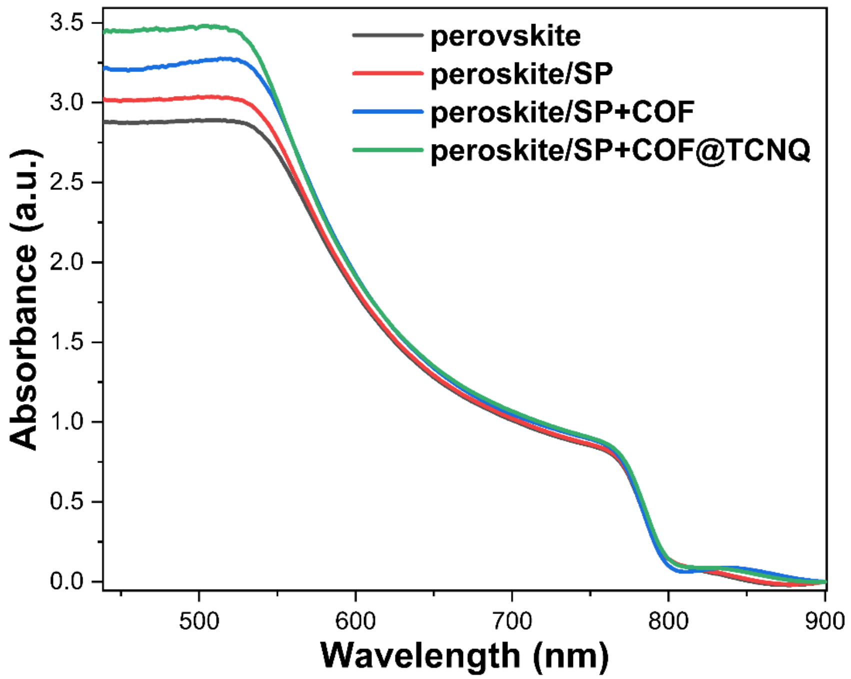

Figure S26. UV-Vis spectra of perovskite film without and with HTL.

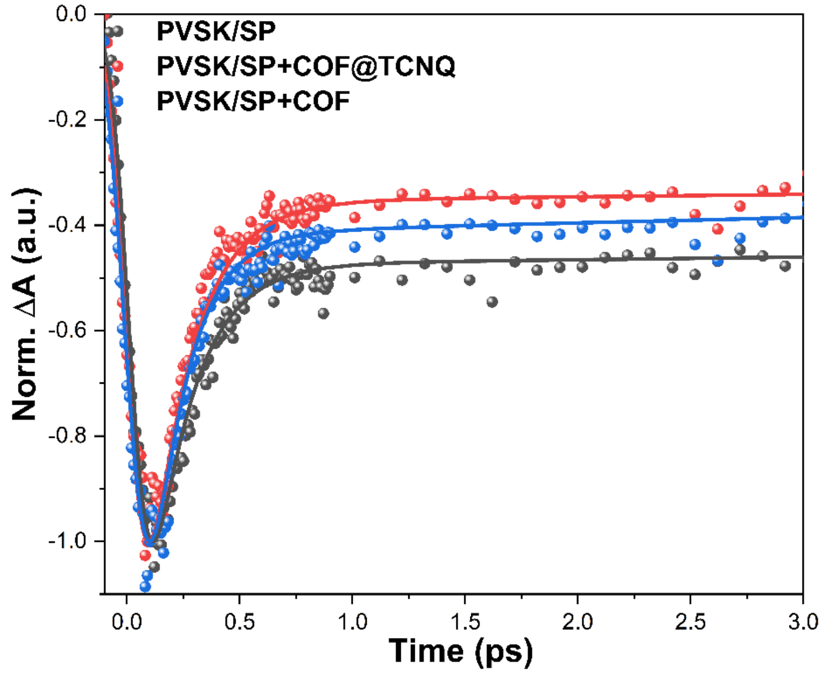

Figure S27. Recovery kinetics of the photobleaching signals for the control, COF@TCNQ-treated and COF-treated samples, excited at 3.10 eV with pump fluence of 5.1 mJ/cm^2.

Table S5. Fitted parameters of the recovery kinetics.

	A_1	t_1 /ps	A_2	t_2 /ps	A_3	t_3 /ps	t_{ave}
**SP**	-0.0032	56.88	-0.0014	705.7	-0.0028	0.18	158.18
**SP+COF@TCNQ**	-0.0029	0.24	-0.0017	332.4	-0.0023	25.53	90.51
**SP+COF**	-0.0022	20.03	-0.0015	0.318	-0.0029	275.01	127.59

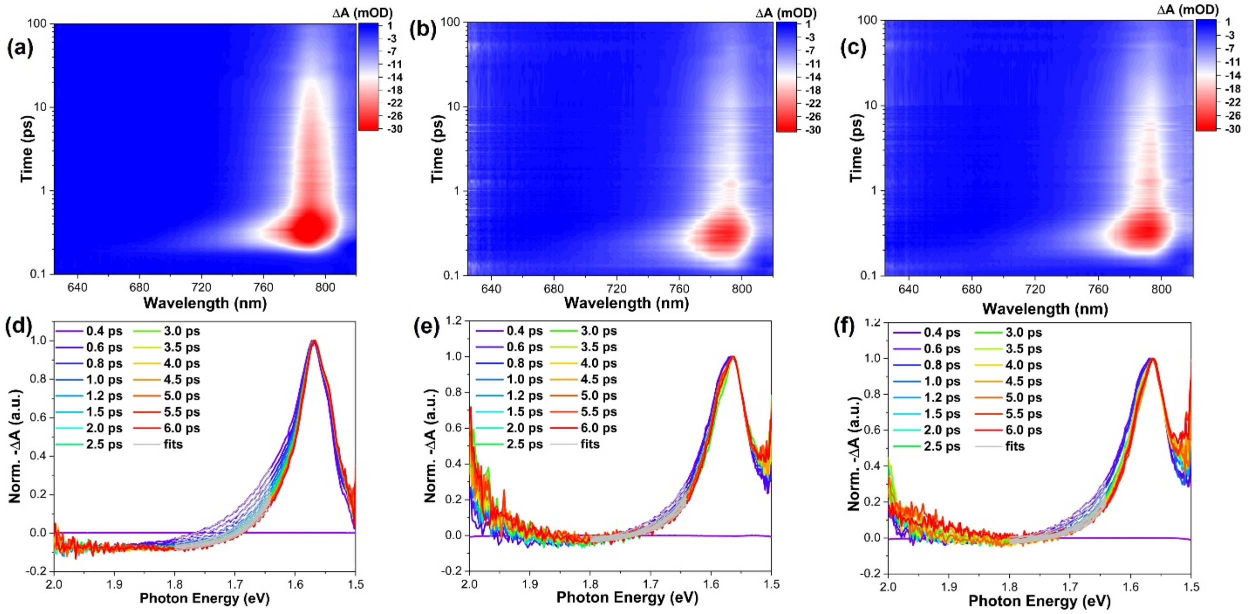

Figure S28. Pseudo-color 2D TA data (top panel) and the corresponding normalized TA spectra (middle panel) pumped at a photon energy of 3.10 eV with pump fluence of 10.2 mJ cm^{-2}. (a, d) Perovskite/Spiro-OMeTAD, (b, e) perovskite/COF@TCNQ-doped Spiro-OMeTAD and (c, f) perovskite/COF-doped Spiro-OMeTAD.

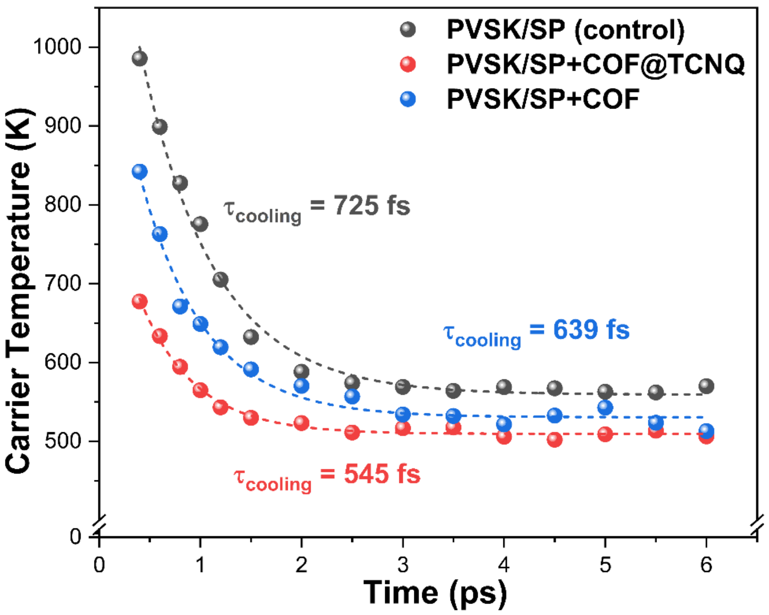

Figure S29. Plots of hot-carrier temperature versus delay time for the perovskite/Spiro-OMeTAD, perovskite/COF@TCNQ-doped Spiro-OMeTAD and perovskite/COF-doped Spiro-OMeTAD films with pump fluence of ~10.2 mJ cm^{-2}.

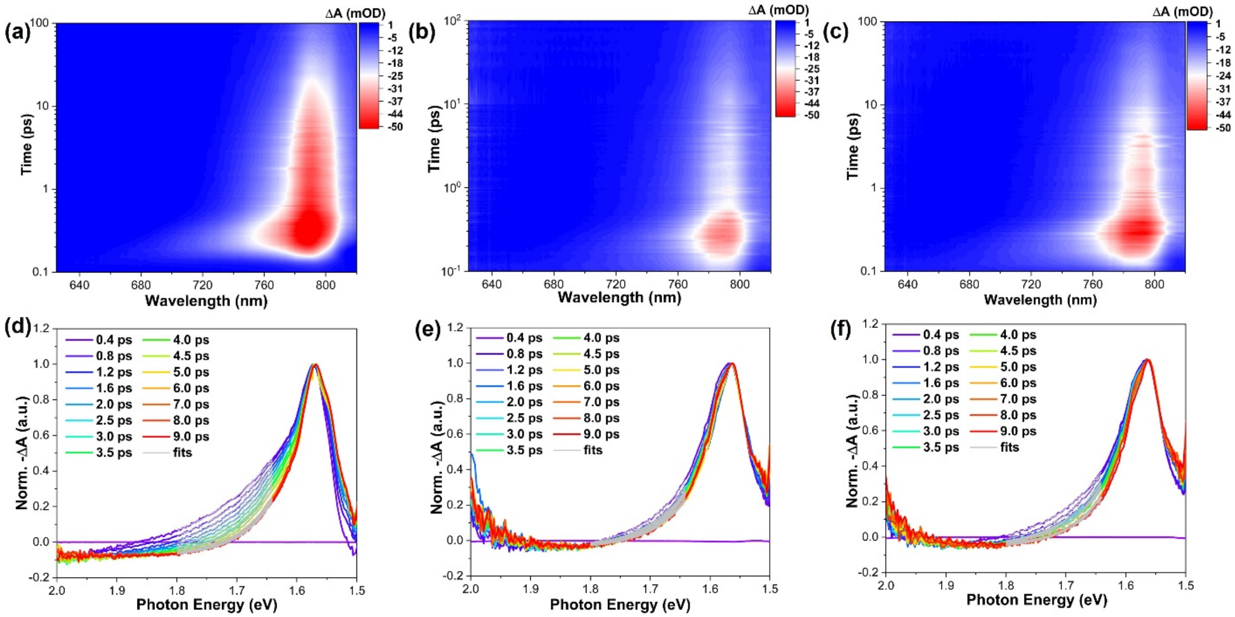

Figure S30. Pseudo-color 2D TA data (top panel) and the corresponding normalized TA spectra (middle panel) pumped at a photon energy of 3.10 eV with pump fluence of 20.4 mJ cm^{-2}. (a, d) Perovskite/Spiro-OMeTAD, (b, e) perovskite/COF@TCNQ-doped Spiro-OMeTAD and (c, f) perovskite/COF-doped Spiro-OMeTAD.

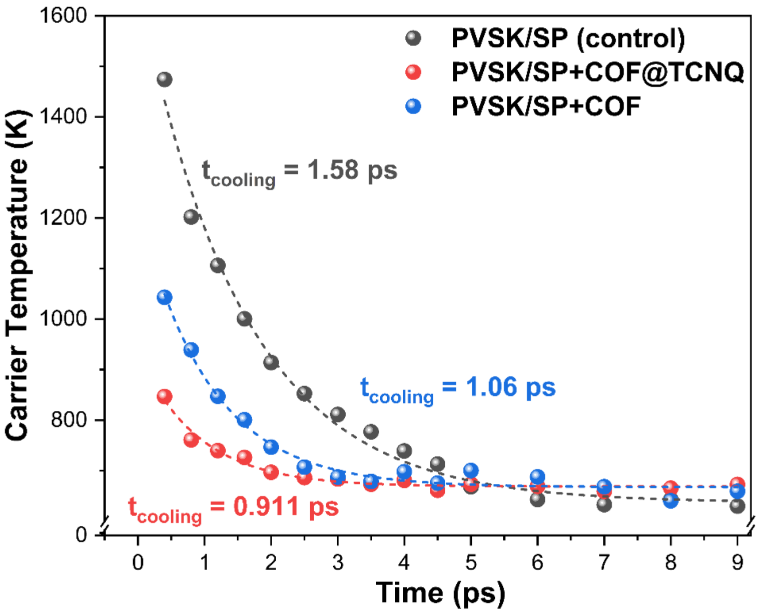

Figure S31. Plots of hot-carrier temperature versus delay time for the perovskite/Spiro-OMeTAD, perovskite/COF@TCNQ-doped Spiro-OMeTAD and perovskite/COF-doped Spiro-OMeTAD films with pump fluence of ~20.4 mJ cm^{-2}.

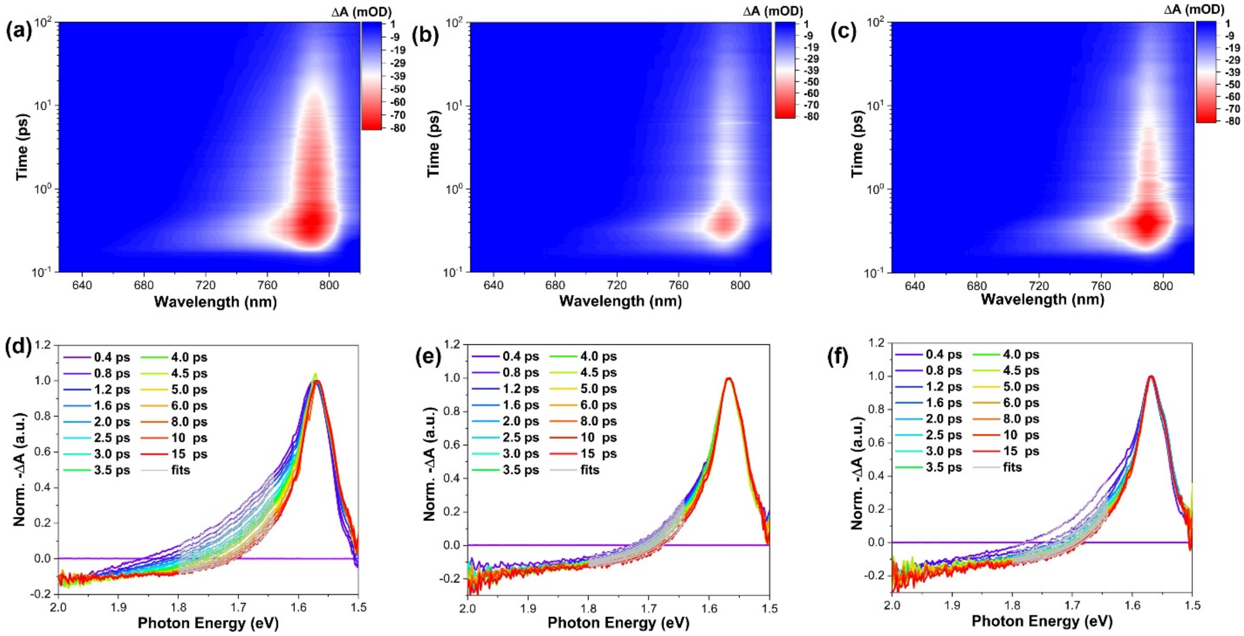

Figure S32. Pseudo-color 2D TA data (top panel) and the corresponding normalized TA spectra (middle panel) pumped at a photon energy of 3.10 eV with pump fluence of 40.8 mJ cm^{-2}. (a, d) Perovskite/Spiro-OMeTAD, (b, e) perovskite/COF@TCNQ-doped Spiro-OMeTAD and (c, f) perovskite/COF-doped Spiro-OMeTAD.

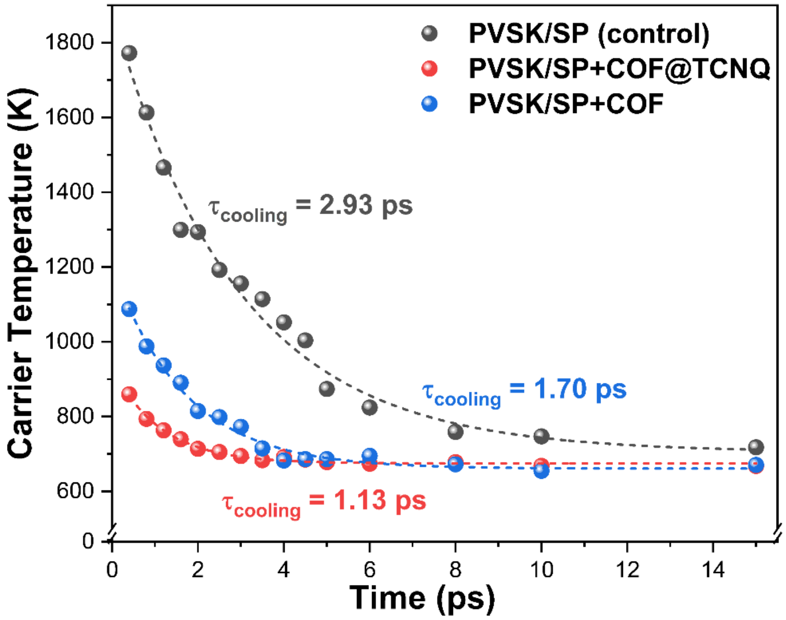

Figure S33. Plots of hot-carrier temperature versus delay time for the perovskite/Spiro-OMeTAD, perovskite/COF@TCNQ-doped Spiro-OMeTAD and perovskite/COF-doped Spiro-OMeTAD films with pump fluence of ~40.8 mJ cm^{-2}.

Supporting references

[S1] Leith, G. A.; Rice, A. M.; Yarbrough, B. J.; Berseneva, A. A.; Ly, R. T.; Buck III, C. N.; Chusov, D.; Brandt, A. J.; Chen, D. A.; Lamm, B. W.; Stefik, M.; Stephenson, K. S.; Smith, M. D.; Vannucci, A. K.; Pellechia, P. J.; Garashchuk, S.; Shustova, N. B. A Dual Threat: Redox-Activity and Electronic Structures of Well-Defined Donor–Acceptor Fulleretic Covalent-Organic Materials. *Angew. Chem. Int. Ed.* **2020**, *59*, 6000–6006.

[S2] Zhao, Y.; Ma, F.; Qu, Z.; Yu, S.; Shen, T.; Deng, H.; Chu, X.; Peng, X.; Yuan, Y.; Zhang, X.; You, J. Inactive $(PbI_2)_2RbCl$ Stabilizes Perovskite Films for Efficient Solar Cells. *Science* **2022**, *377*, 531–534.

[S3] Li, H.; Chang, J.; Li, S.; Guan, X.; Li, D.; Li, C.; Tang, L.; Xue, M.; Yan, Y.; Valtchev, V.; Qiu, S.; Fang, Q. Three-Dimensional Tetrathiafulvalene-Based Covalent Organic Frameworks for Tunable Electrical Conductivity. *J. Am. Chem. Soc.* **2019**, *141*, 13324−13329.

[S4] Ji, X.; Zhou, T.; Fu, Q.; Wang, W.; Wu, Z.; Zhang, M.; Guo, X.; Liu, D.; Woo, H. Y.; Liu, Y. Dopant-Free Two-Dimensional Hole Transport Small Molecules Enable Efficient Perovskite Solar Cells. *Adv. Energy Mater.* **2023**, *13*, 2203756.

[S5] Torabi, S.; Jahani, F.; Severen, I. V.; Kanimozhi, C.; Patil, S.; Havenith, R. W. A.; Chiechi, R. C.; Lutsen, L.; Vanderzande, D. J. M.; Cleij, T. J.; Hummelen, J. C.; Koster, L. J. A. Strategy for Enhancing the Dielectric Constant of Organic Semiconductors Without Sacrificing Charge Carrier Mobility and Solubility. *Adv. Funct. Mater*. **2015**, *25*, 150–157.

[S6] Wu, J.; Li, M.; Fan, J.; Li, Z.; Fan, X.; Xue, D.; Hu, J. Regioselective Multisite Atomic-Chlorine Passivation Enables Efficient and Stable Perovskite Solar Cells. *J. Am. Chem. Soc*. **2023**, *145*, 5872–5879.
